# Sequential syntax of EEG microstates captures the temporal dynamics of inhibitory control

**DOI:** 10.1016/j.isci.2026.118097

**Published:** 2026-11-03

**Authors:** Omar Cano-Garcia, Keith Guzmán-Díaz, Sergio Rivera-Tello, Hugo Vélez-Pérez, J. Alejandro Morales, Julieta Ramos-Loyo, Rebeca Romo-Vázquez, Omar Paredes

**Affiliations:** 1 Universidad de Guadalajara, Centro Universitario de Ciencias Exactas e Ingenierías, Departamento de Bioingeniería Traslacional, Biodigital Innovation Lab, Guadalajara, Jalisco, México; 2 Universidad de Guadalajara, Centro Universitario de Ciencias Exactas e Ingenierías, Departamento de Bioingeniería Traslacional, Guadalajara, Jalisco, México; 3 Universidad de Guadalajara, Centro Universitario de Ciencias Biológicas y Agropecuarias, Instituto de Neurociencias, Guadalajara, Jalisco, México

**Keywords:** EEG microstates, microstate syntax, conditional successor analysis, multiple sequence alignment, inhibitory control, sequential brain dynamic

## Abstract

Electroencephalogram (EEG) microstates are successive global brain states whose temporal organization may reflect cognitive dynamics that conventional summary statistics do not capture. We applied a sequence-based framework, combining conditional successor analysis with multiple sequence alignment adapted from genomics, to microstate sequences recorded during a Go/NoGo task in 30 healthy adults. Generalized estimating equations showed no condition-level differences in standard microstate metrics. Conditional successor analysis identified three motifs enriched in the NoGo-early window (100–500 ms): A-C-D (*p*_FDR_ = 0.013), B-A-D (*p*_FDR_ = 0.029), and E-A-D (*p*_FDR_ = 0.040), all passing through microstate A and ending in microstate D, consistent with fronto-central engagement. Multiple sequence alignment showed non-random temporal organization in all conditions (all *z* > 11.5 and *p* < 0.001). These results support sequence syntax as a framework for linking millisecond-scale brain dynamics to cognitive function.

## Highlights

• EEG microstate sequences during Go/NoGo reveal condition-specific sequential motifs
• Three NoGo-enriched motifs (A-C-D, B-A-D, and E-A-D) emerge in the early processing window
• Sequential syntax, not static metrics, captures inhibitory control dynamics in EEG
• MSA reveals a shared temporal architecture aligned with the N2 inhibitory window

## Graphical abstract

**Figure:**
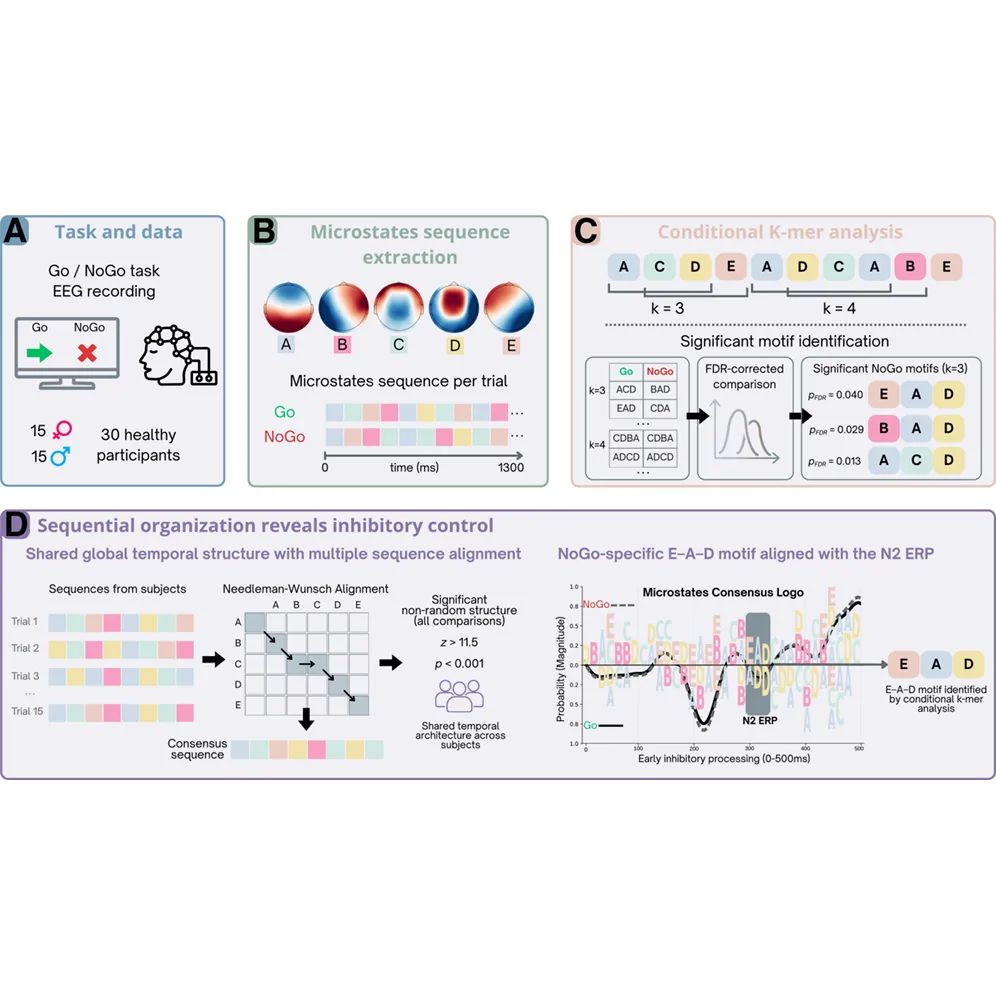

## Teaser

Neuroscience; Cognitive neuroscience; Computational biology

## Introduction

Classical electroencephalogram (EEG) microstate research has relied predominantly on static parameters such as duration, occurrence, and coverage to characterize the four canonical topographic maps (A–D) that segment continuous brain activity.^1^ While these metrics reliably differentiate cognitive states and clinical populations, they provide only a coarse description of neural dynamics and overlook the temporal organization of microstates as state succession.^2^^,^^3^ Crucially, they reveal how often microstates appear but not how they assemble into temporal sequences, leaving recurrent motifs, transition loops, and higher order patterns largely hidden.

Recent work has shifted the field toward dynamic characterizations of microstate structure. Evidence now shows that microstate transitions are non-Markovian—meaning that the probability of a given transition depends on the recent history of states rather than solely on the current configuration—exhibit long-range dependencies, and encode temporally structured patterns referred to as microstate syntax.^4^^,^^5^ Metrics based on entropy, auto-information, and scale-free properties demonstrate that microstate sequences preserve information over hundreds of milliseconds and differ systematically across cognitive conditions and levels of consciousness.^6^

A recent comprehensive review underscores that microstate syntax is emerging as a central but methodologically challenging frontier in microstate research.^7^ Key methodological issues—including differences between event-mode and clock-mode segmentation, sensitivity to backfitting parameters, and distortions introduced by winner-take-all labeling—must be considered when interpreting temporal patterns. Importantly, this review highlights growing interest in modeling microstate sequences with tools inspired by linguistics and computational biology, including n-grams, causal-state models, and other sequence-based approaches, while noting that integrated applications to cognitive paradigms remain scarce.

Despite this accelerating methodological innovation, a critical gap persists: existing approaches largely characterize *marginal* properties of microstate sequences—how often transitions occur, or how frequently a given motif appears—without isolating the *conditional* structure that determines which state follows a given transition.^8^ This distinction is particularly consequential for inhibitory control, a temporally ordered sequence of cognitive operations spanning early attentional processes to late response evaluation, where the relevant question is not merely whether a transition occurs but which trajectory the system selects at each branching point. Long-range temporal dependencies confirm that such dynamics cannot be reduced to simple pairwise transitions,^9^^,^^10^ and alterations in the temporal parameters of canonical microstate C have been directly linked to impaired inhibitory control in Go/NoGo paradigms.^11^ Crucially, recent evidence demonstrates that the cost of transitioning between microstates scales with cognitive control demands and predicts behavioral performance in conflict tasks,^12^ revealing a level of dynamical organization that remains inaccessible to conventional static descriptors or marginal transition probabilities.

The present study addresses this gap through two complementary sequence-based approaches adapted from genomics. First, we introduce a conditional successor analysis within a k-mer syntax framework, which isolates the identity of the state following each directed transition—decoupling the decisional component of sequence dynamics from the marginal frequency of individual transitions. Second, we apply multiple sequence alignment (MSA) using the Needleman-Wunsch algorithm to quantify global trajectory similarity across trials and conditions, enabling detection of shared temporal architecture at the population level and testing whether condition-specific differences reflect local bifurcations within a common global structure or wholesale reorganization of sequential dynamics.

Together, these methods treat microstate sequences as symbolic trajectories and test two specific hypotheses; (1) that condition-specific motifs emerge in early processing phases through bifurcations in conditional transition dynamics rather than through differences in marginal motif frequencies; and (2) that Go and NoGo trials share a common global temporal architecture, with inhibitory control expressed through local sequential patterns rather than through global sequence reorganization.

## Results

An overview of the complete analysis pipeline is illustrated in Figure 1. The workflow comprises four main stages: (1) data acquisition and preprocessing, (2) microstate analysis with extraction of conventional metrics, (3) syntax extraction through k-mer pattern analysis, and (4) sequence alignment and group comparison using bioinformatics-derived algorithms.

**Figure 1:**
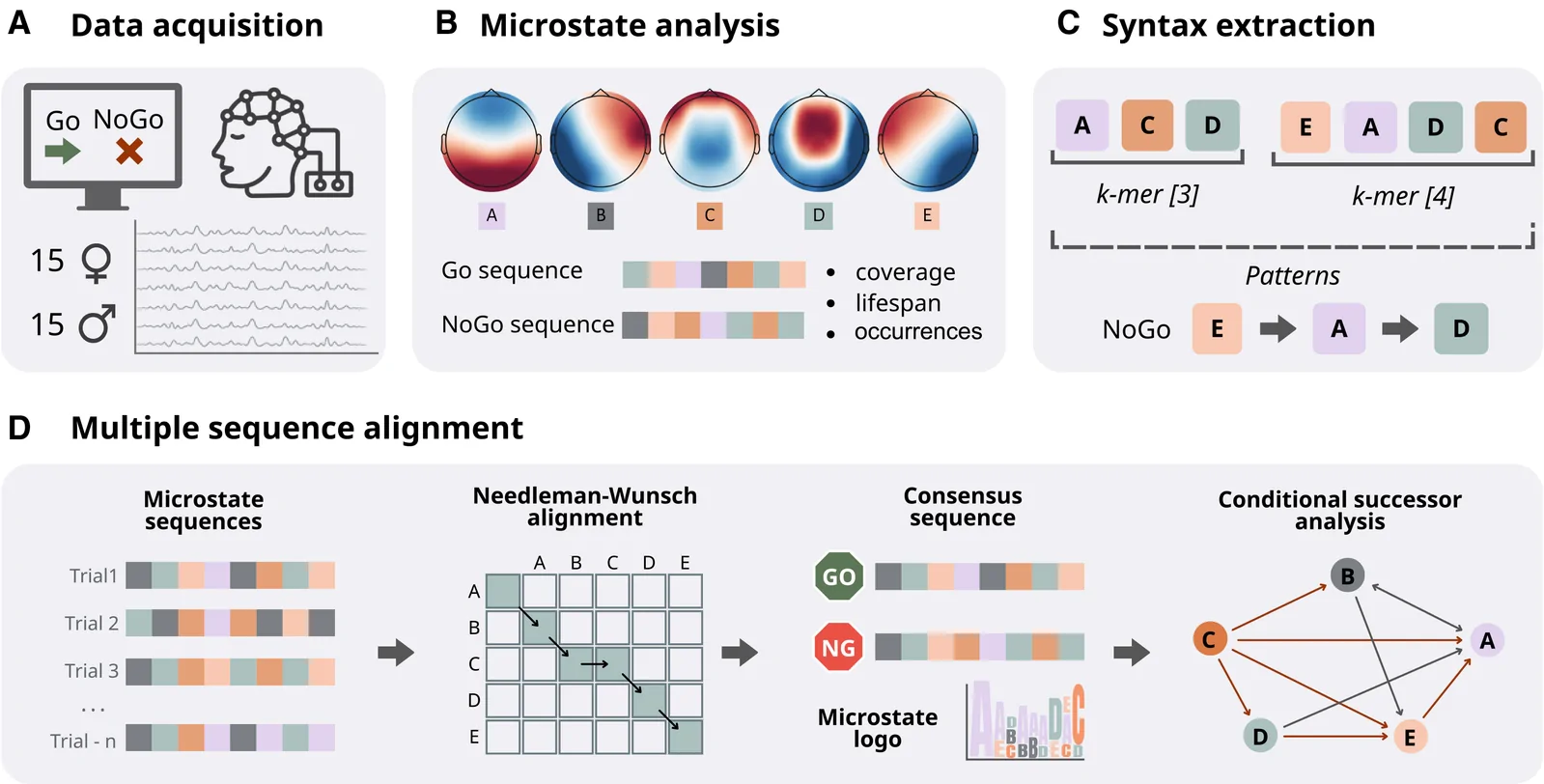
Overview of the EEG microstate and sequence analysis pipeline (A) Data acquisition from 30 adults performing a Go/NoGo task, followed by preprocessing (average reference, 4–30 Hz bandpass filter). (B) Microstate analysis yields five topographies (A–E) and conventional metrics (coverage, duration, and occurrence). (C) Syntax extraction converting continuous EEG into discrete microstate sequences, with k-mer analysis (k = 3, 4, 5) to identify recurring patterns. (D) Sequence alignment using Needleman-Wunsch algorithm for group comparison (Go vs. NoGo) with subsequent statistical analysis.

### Microstate computation and selection

Clustering analyses were performed for K = 4–9. Although global explained variance (GEV) increases monotonically with K—a known property of K-means algorithms—the optimal solution was determined using three convergent criteria applied simultaneously: GEV threshold, topographic independence, and physiological plausibility. Although GEV continued to increase beyond K = 6 (Figure 2A), the six-microstate solution (GEV = 58.3%) was selected as the first to reach the 58% benchmark reported for resting-state data^1^; however, inspection of its topographies revealed one microstate with a noise-like distribution, characterized by activity concentrated almost exclusively over the right temporal electrode (T4). This atypical map—and the full set of six microstates—is shown in Figure S2. Moreover, the spatial correlation matrix for K = 6 displayed strong pairwise correlations among microstates D, E, and F (microstates were labeled A–F in order of appearance from the clustering procedure; |r| = 0.62–0.68; Figure 2B), indicating substantial topographical redundancy. These findings—artifact-like morphology and diminished separability—led to the exclusion of the K = 6 solution. Additional spatial correlation matrices and microstate topographies for K = 4–9 are provided in Figure S3.

**Figure 2:**
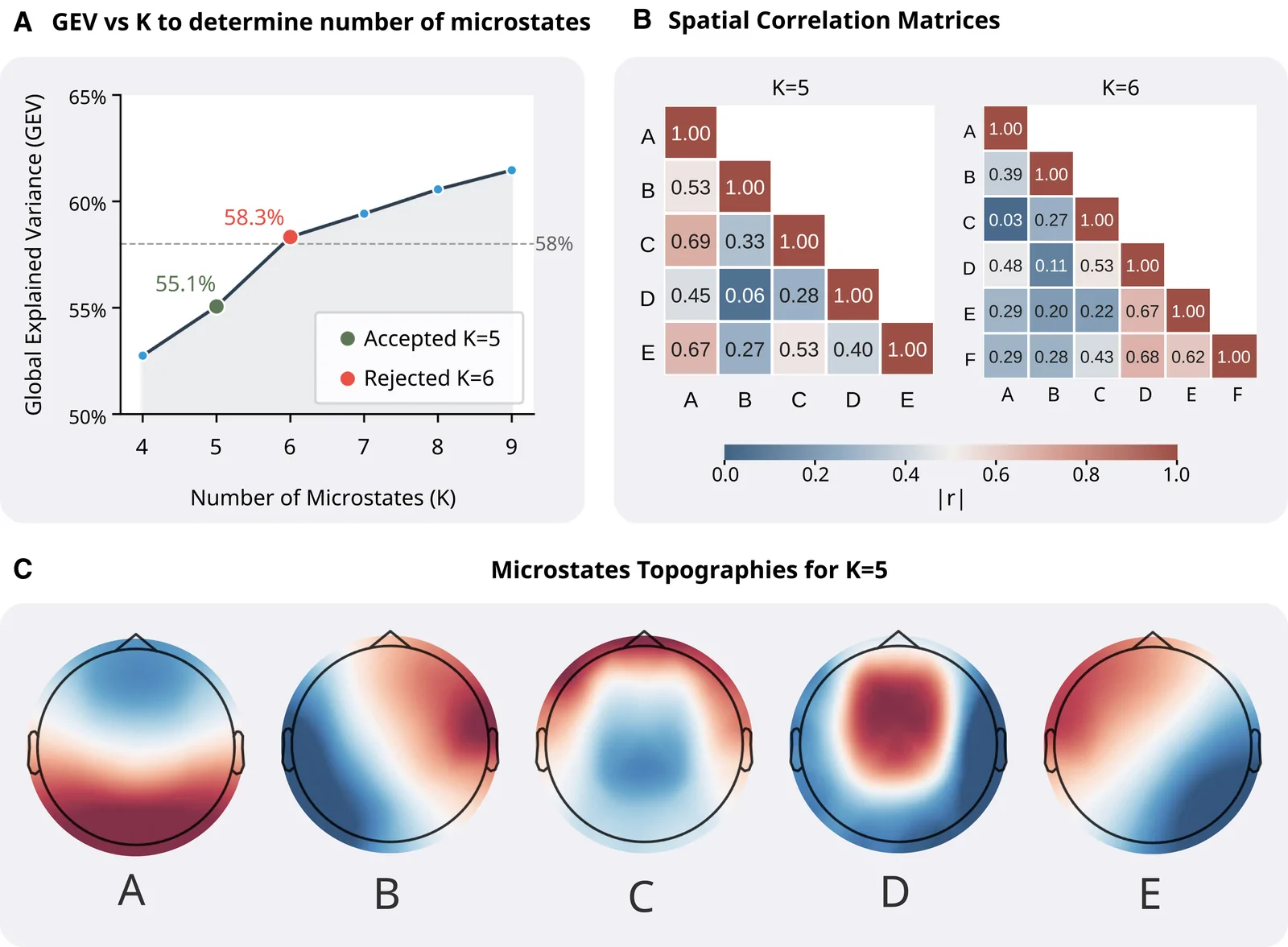
Selection of the optimal number of microstates (A) Global explained variance (GEV) as a function of the number of microstates (K), K = 5 (green) was accepted over K = 6 (red) based on parsimony and neurophysiological plausibility. (B) Spatial correlation matrices for K = 5 and K = 6 solutions. Note the high correlations among microstates D, E, and F in the K = 6 solution (|r| = 0.62–0.68), indicating topographical redundancy. (C) Scalp topographies for the five selected microstates (A–E), showing spatially distinct configurations with interpretable neural generators.

Given these limitations, we selected the five-microstate solution (K = 5; microstates were labeled A–E in order of appearance from the clustering procedure), which achieved a GEV of 55.1%—a value expected for task-locked epochs, where increased variability typically lowers explained variance relative to resting-state benchmarks.^1^ Although microstate A showed bilateral spatial correlations with microstates C and E (Figure 2B), these do not constitute the triadic redundancy pattern that characterized the rejected K = 6 solution, and the five topographies remain spatially distinguishable (Figure 2C). Their correspondence with canonical microstate templates across three independent reference sets is reported in Table 1 and detailed in Data S1; functional associations are inferred from prior network literature.

**Table 1:** Microstate labels, scalp topographies, canonical correspondences, and associated functions for the K = 5 solution

Label	Topography	Canonical correspondence (|r| × 100)	Functional association
A	symmetric anterior-posterior, frontal +	Koenig et al.^13^ C (97.1%, inv.), Milz et al.^14^ D (99.2%, inv.), Artoni et al.^15^ C (97.3%, inv.)	default mode/visual networks^1^^,^^16^
B	right frontal to left posterior (diagonal)	Koenig et al.^13^ A (87.7%), Milz et al.^14^ A (94.8%), Artoni et al.^15^ A (95.8%)	auditory processing^1^^,^^16^
C	fronto-parietal	Artoni et al.^15^ E (94.0%, inv.)^a^	task-specific processing^15^^,^^17^
D	fronto-central	Koenig et al.^13^ D (77.6%), Artoni et al.^15^ D (92.2%)	executive function^1^^,^^14^
E	left frontal to right posterior (diagonal)	Koenig et al.^13^ B (94.1%), Milz et al.^14^ B (98.5%), Artoni et al.^15^ B (94.9%)	visual processing/attention^17^^,^^18^

Canonical correspondences are based on polarity-invariant spatial similarity (|r| × 100) against three reference sets. Functional associations are inferred from prior literature (for more information see Data S1).

inv. = inverse polarity (signed r < 0); polarity is ignored in similarity computation per standard microstate methodology.^1^ Functional associations are inferred from spatial correspondence with canonical maps and prior network literature; they are not directly validated in this dataset. Similarity values represent polarity-invariant spatial similarity (|r| × 100) after restriction to the common 19-channel montage.

a Under one-to-one assignment against the four-map references (K = 5 > K = 4), microstate C is unassigned against Koenig et al.^13^, and microstate D is unassigned against Milz et al.^14^ (C is assigned to Milz et al.^14^ C, 87.4%); its highest match is Artoni et al.^15^ E (94.0%), identifying it as the fifth canonical class not represented in four-class solutions. See Figure S6 for complete similarity matrices.

All five microstates were consistently observed across participants and trials (per-trial values for all five microstates in Table S1). Each microstate appeared in ≥95% of trials in at least 28 of 30 participants—including microstate C, which has no direct equivalent in canonical four-map resting-state solutions—confirming that all five classes reflect robust participant-level phenomena rather than subset-driven artifacts.

### Assessment of sex bias and condition effect in microstate metrics

Generalized estimating equations (GEEs) were used to assess whether microstate metrics differed by sex and by task condition (Go vs. NoGo). For each metric, a full model (including sex and condition × sex interaction) was compared against a reduced model (excluding sex terms) to evaluate potential sex-related bias (see Table S1). A summary of test statistics and decisions is provided in Table 2.

**Table 2:** GEE model comparison for sex bias and condition effects across microstate metrics

Metric	Wald *p* (full)	Wald *p* (reduced)	ΔAIC	ΔQIC	Sex effect	Condition effect
Coverage	>0.99	>0.99	+4.0	+0.115	no	no
Lifespan	0.204	0.766	−194.6	−0.756	no	no
Occurrences	0.074	0.273	−192.8	−200.5	no	no

For each metric, a full model (metric ∼ condition + microstate + sex + condition:sex) was compared against a reduced model (metric ∼ condition + microstate). Positive ΔAIC and ΔQIC values favor the reduced model (no sex); negative values favor the full model (with sex). Wald *p* (condition) was derived from the reduced model. All tests used an exchangeable working correlation structure. Coverage sums to 1 across microstates in each trial; its condition and sex terms are therefore null by construction (*p* > 0.99).

No significant sex effects were detected across any of the three metrics (all Wald *p* > 0.05; Table 2). For coverage, all criteria consistently supported the reduced model: the Wald test was non-significant (*p* > 0.99), delta Akaike information criterion (ΔAIC) favored exclusion of sex (+4.0), and delta quasi-likelihood under the independence model criterion (ΔQIC) was negligible (+0.115). For lifespan, the Wald test was non-significant (*p* = 0.204) and ΔQIC did not meet the inclusion threshold (−0.756), despite a numerically large ΔAIC (−194.6); this discrepancy likely reflects the sensitivity of AIC to sample size in gamma-distributed outcomes, and model selection was therefore guided by the Wald statistic and ΔQIC. For occurrences, the Wald test was not significant (*p* = 0.074); although ΔAIC and ΔQIC strongly favored the full model (−192.8 and −200.5, respectively), the absence of a statistically significant Wald test led to the exclusion of sex from the final model. Accordingly, sex was excluded from all final models. Model comparison for all three metrics is summarized in Figure S4.

Condition effects were likewise non-significant across all three metrics (coverage: *p* > 0.99; lifespan: *p* = 0.766; occurrences: *p* = 0.273; Table 2), indicating that neither the proportion of time dominated by each microstate, its mean duration, nor its occurrence rate differed meaningfully between Go and NoGo trials.

The absence of both sex-related bias and condition-level effects across conventional microstate metrics suggests that these global descriptors lack sensitivity to the temporal dynamics underlying inhibitory control. This motivated the use of sequence-based analyses, described below, to capture condition-related differences emerging from higher order patterns of neural state transitions rather than from single-microstate properties.

### Condition-specific motifs in microstate syntax

Motif analysis was conducted on the full 1,300 ms epoch and subsequently on the early (100–500 ms) and late (500–900 ms) temporal windows.

#### Exhaustive k-mer screen

An agnostic screen of all k-mer motifs (k = 3, 4, and 5) yielded no significant results after false discovery rate (FDR) correction in any temporal window (full results in Table S2). Across the 1,680 motifs tested per window (80 three-mers, 320 four-mers, and 1,280 five-mers in the full epoch; 1,677 and 1,679 in the early and late windows, respectively, because motifs with fewer than five occurrences were excluded), the number of uncorrected significant motifs (*p* < 0.05, before FDR correction) was consistent with chance expectation, indicating that gross distributional differences in motif frequencies do not distinguish Go from NoGo conditions at the marginal level.

To assess the empirical stability of the three FDR-significant motif frequencies identified in the early phase (A-C-D, B-A-D, and E-A-D), a split-half reliability analysis was conducted. Each participant’s 20 NoGo trials were divided into two non-overlapping halves (trials 1–10 vs. 11–20); mean motif rates were computed per half per participant and correlated across participants using Pearson’s r, with full-test reliability estimated via the Spearman-Brown correction. Motifs E-A-D (r = 0.469, Spearman-Brown ρ = 0.639, and *p* = 0.009), A-C-D (r = 0.428, ρ = 0.599, and *p* = 0.018), and B-A-D (r = 0.411, ρ = 0.583, and *p* = 0.024) showed moderate split-half reliability. These values reflect the aggregate nature of the analysis (motif counts pooled across 600 trials per condition) and are reported as a stability benchmark rather than a predictor of individual-trial responses.

#### Transition-level analysis

Bigram rates for all 20 directed microstate transitions were compared between conditions using Mann-Whitney U tests (uncorrected *p* values and Benjamini-Hochberg FDR across the 20 bigrams per window and level, α = 0.05; see STAR Methods). At trial level, two bigrams differed between conditions at the uncorrected level in the early phase: A → D (*p* = 0.021, *p*_FDR_ = 0.222, and r = 0.067) and C → E (*p* = 0.022, *p*_FDR_ = 0.222, r = 0.066), neither survived FDR correction. No bigram survived FDR correction at either level in any window (full results in Table S3). Critically, the E → A bigram—the prefix of the E-A-D motif—showed no difference between conditions (early phase: *p* = 0.899, *p*_FDR_ = 0.899, and r = 0.004), providing no evidence of a difference in the rate at which the system initiates the E → A transition between Go and NoGo trials.

#### Conditional successor analysis

To isolate the decisional component of microstate transitions, we tested whether the identity of the successor state following each bigram differed between conditions. For each of the 20 directed bigrams, Fisher’s exact tests (one-sided, NoGo enrichment) were applied to each possible successor, with FDR correction within each bigram’s successor set (4 tests per bigram). This conditional framing revealed three motifs surviving FDR correction in the early phase (100–500 ms): A-C-D (*p*_FDR_ = 0.013), B-A-D (*p*_FDR_ = 0.029), and E-A-D (*p*_FDR_ = 0.040), all enriched in NoGo (Table 3). Outside the early window, the full epoch yielded A-B-E (*p*_FDR_ = 0.006), C-B-D (*p*_FDR_ = 0.0496) and D-E-B (*p*_FDR_ = 0.037), and the late window D-E-B (*p*_FDR_ = 0.046) (Table S4); none involved the E-A-D sequence.

**Table 3:** Conditional successor motifs with significant NoGo enrichment in the early phase (100–500 ms)

Bigram	Motif	Go *n* (%)	NoGo *n* (%)	Diff	OR	*p*	*p*_FDR_	Sig
A → C	A-C-D	163 (19.2%)	206 (24.8%)	+5.6	1.388	0.003	0.013	✓
B → A	B-A-D	175 (22.5%)	216 (28.0%)	+5.5	1.341	0.007	0.029	✓
E → A	E-A-D	197 (24.1%)	245 (29.2%)	+5.2	1.304	0.010	0.040	✓

For each directed bigram (M_1_M_2_), the conditional proportion P(X |M_1_ → M_2_) was compared between Go and NoGo using Fisher’s exact test (one-sided). FDR correction (Benjamini-Hochberg) was applied within each bigram’s successor set. OR > 1 indicates NoGo enrichment. Only motifs surviving FDR <0.05 are shown; full results across all windows in Table S4.

This pattern of results demonstrates that condition-specific sequential structure is not detectable at the level of marginal motif frequencies or individual transition rates, but emerges when the analysis is conditioned on specific preceding transitions—isolating the bifurcation point at which the NoGo condition selectively channels microstate dynamics toward inhibitory states. The E-A-D motif was absent in the late phase (500–900 ms), confirming temporal specificity broadly consistent with the N2/P3 inhibitory complex. At the trial level, microstate D also occurred more frequently in NoGo trials during the early window (Go: 0.204 ± 0.132 vs. NoGo: 0.214 ± 0.115; *p* = 0.014 and r = 0.071), although this effect did not survive aggregation to the subject level (*p* = 0.102).

### Temporal structure of microstate sequences revealed by MSA

To validate that subject-level consensus sequences encode genuine temporal organization rather than reflecting microstate frequency alone, pairwise between-subject Needleman-Wunsch similarity was computed within each condition and temporal window (consensus sequences in Table S5) and tested against permutation-based null distributions.

All four cells showed observed mean similarity significantly exceeding their shuffled counterparts (Go-early: 0.333, Go-late: 0.343; NoGo-early: 0.341, NoGo-late: 0.349; null range: 0.268–0.278; all *z* > 11.5, all *p* < 0.001). At the pair level, 72%–77% of between-subject scores exceeded the null median (group-level odds ratio [OR] = 2.60–3.47, Fisher’s exact *p* < 0.001; Figure 3). At the subject level, 30/30 Go and 29/30 NoGo participants exhibited individually significant similarity relative to their shuffled baselines (binomial *p* < 0.001 in all cells). These results confirm that the temporal ordering of microstate sequences carries shared structures across individuals that cannot be attributed to state-frequency composition.

**Figure 3:**
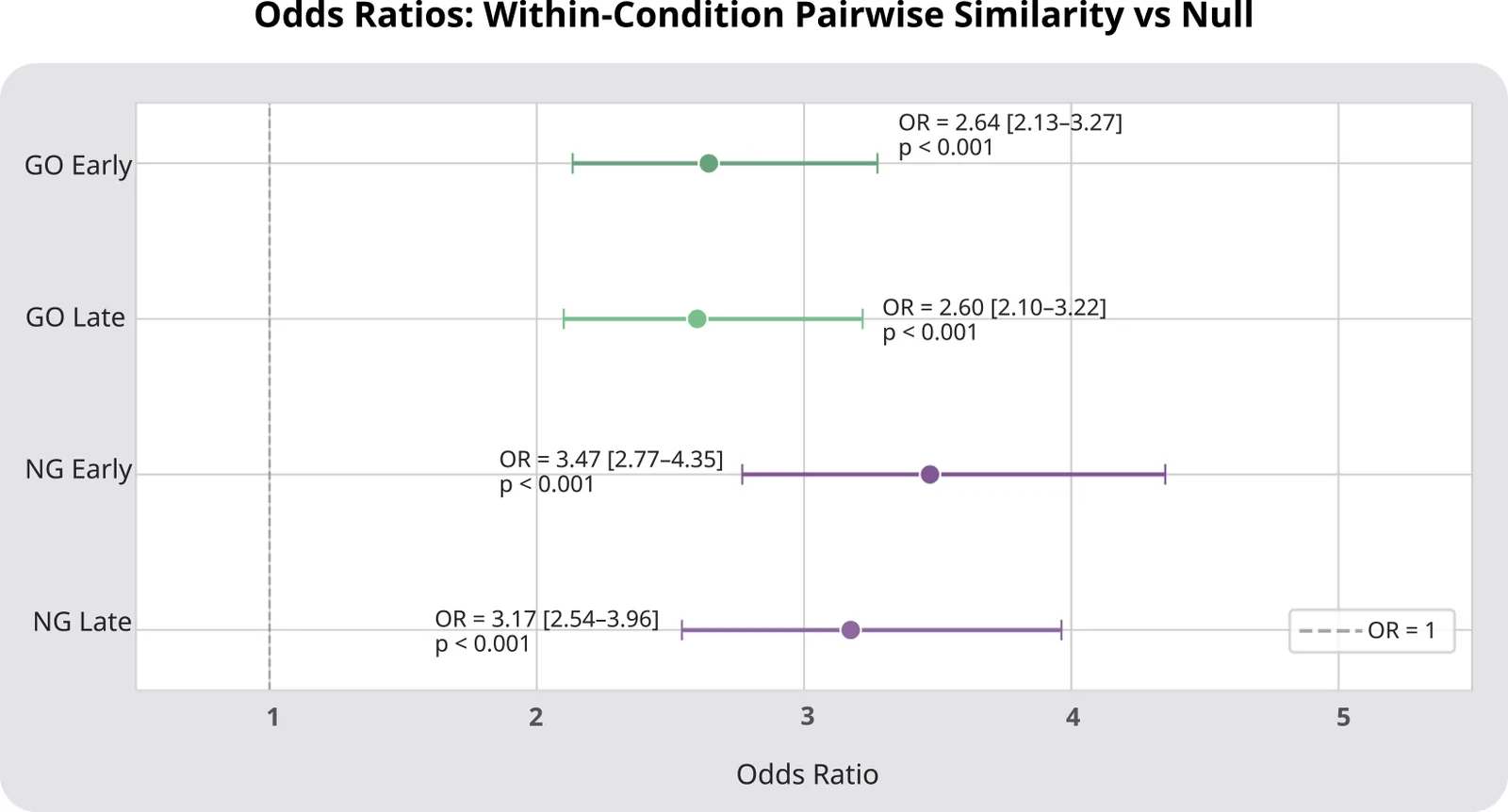
Forest plot of group-level odds ratios for within-condition pairwise sequence similarity versus shuffled null distributions, computed separately for early (100–500 ms) and late (500–900 ms) temporal windows Each point represents the odds ratio comparing the proportion of between-subject Needleman-Wunsch alignment scores exceeding the null median (observed vs. shuffled sequences) for a given condition and temporal window. Error bars indicate 95% confidence intervals. Green: Go condition; purple: NoGo condition. All four cells significantly exceeded chance (Fisher’s exact *p* < 0.001), confirming that subject-level consensus sequences encode non-random temporal structure independent of microstate frequency composition.

Although all four cells showed comparable departure from their respective null distributions, the group-level ORs revealed a directionally consistent pattern (Figure 3): NoGo conditions yielded higher ORs than Go in both temporal windows (NoGo-early: OR = 3.47 [2.77–4.35] vs. Go-early: OR = 2.64 [2.13–3.27]; NoGo-late: OR = 3.17 [2.54–3.96] vs. Go-late: OR = 2.60 [2.10–3.22]), suggesting greater relative sequential structure in NoGo sequences. Within NoGo, the early window showed a higher OR than the late window (3.47 vs. 3.17), whereas Go ORs were virtually identical across windows (2.64 vs. 2.60), suggesting that the additional sequential constraint in NoGo is concentrated in the early processing phase. Although overlapping confidence intervals preclude formal statistical inference on these differences, the pattern is consistent with stronger local sequential constraint during early NoGo processing—the same window in which the three condition-specific motifs were identified by conditional successor analysis.

Similarity values were otherwise comparable across all four cells (range: 0.333–0.349), indicating that both Go and NoGo trials share a common global temporal architecture throughout the epoch. The qualitative differences visible in the logo plots—where Go sequences exhibit more rhythmic, cyclic patterns (e.g., B-C loops) whereas NoGo sequences display greater variability with increased involvement of microstate A—thus reflect differences in the specific configuration of shared temporal structure rather than the presence versus absence of structure itself.

Logo plots derived from the meta-consensus sequences further illustrate these temporal reorganizations (Figure 4). Positions shown in gray correspond to sequence locations where length variability across one or two individuals produced insufficient coverage for reliable consensus estimation and are excluded from interpretation. The first ∼33 consensus positions correspond to the early phase (100–500 ms), and positions ∼45–75 correspond to the late phase (500–900 ms). During the early phase, the consensus sequences of both conditions include the E-A-D sequence, temporally coinciding with the N2 component (peaking at ∼218 ms at Fz) previously documented in this dataset and visible in the event-related potential (ERP) traces (Figure 4). During the late phase, the consensus structure diverges between conditions: Go sequences exhibit repetitive B-C patterns, while NoGo sequences display more variable configurations extending into the window of the P3 component (∼280–500 ms at Fz; Figure 4). The full-epoch consensus logo plot is provided in Figure S5. These plots are descriptive; the condition difference in E-A-D is established by the conditional successor analysis (Table 3).

**Figure 4:**
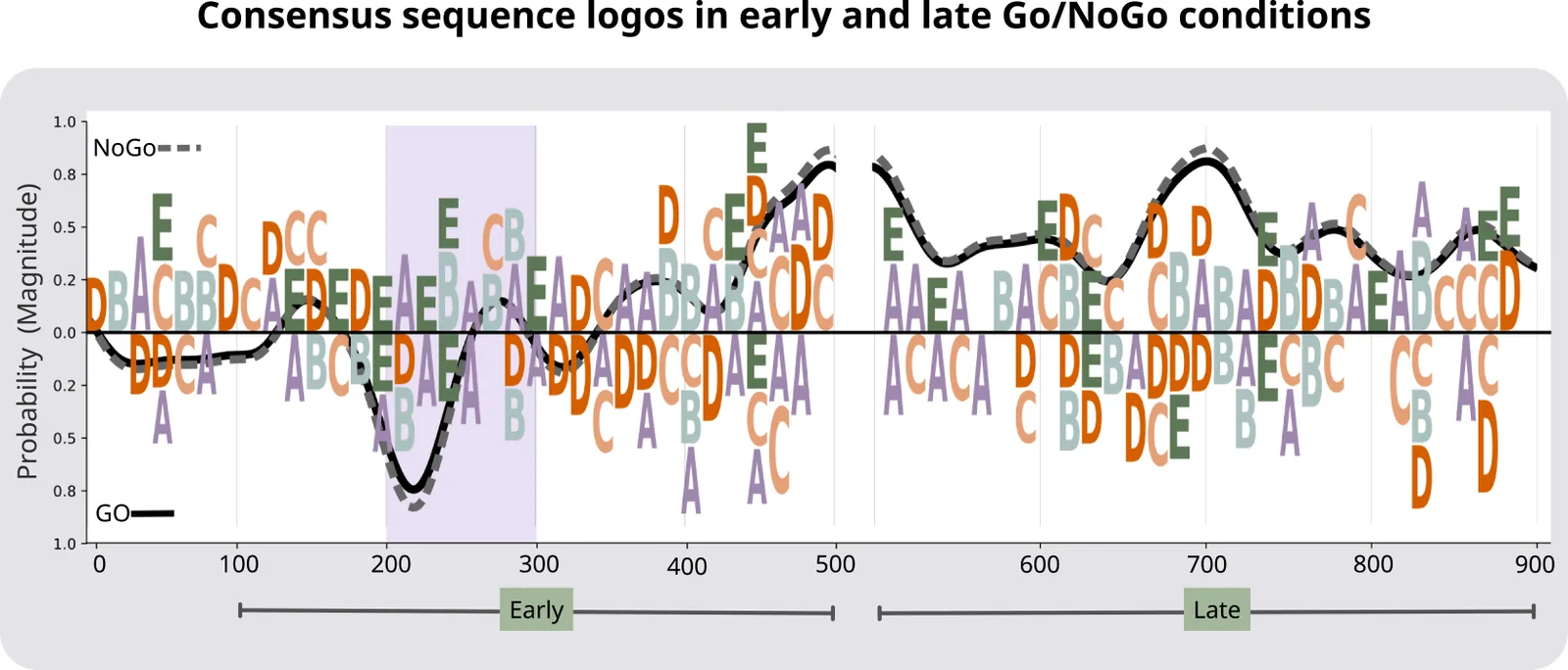
Consensus sequence logo plots for NoGo and Go conditions across early (100–500 ms) and late (500–900 ms) phases Letter height indicates the probability of each microstate at a given consensus position. Only microstates reaching a representation threshold of ≥25% were retained in the logo. The purple shaded region highlights the N2 window, with the ERP waveform (Go: solid black line; NoGo: dashed gray line) shown for temporal reference. The logos are a descriptive visualization of the consensus sequences and do not test differences between conditions; statistical evidence for NoGo enrichment of E-A-D comes from the conditional successor analysis; NoGo: top and Go: bottom (Table 3).

## Discussion

The present results reveal that inhibitory control is expressed through the sequential syntax of microstate transitions rather than through changes in individual state properties, operating at two complementary levels: condition-specific bifurcation motifs during the early processing phase and a shared global temporal architecture present across all conditions and windows. The convergence of conditional successor analysis and sequence alignment reveals a dual-level organization: a shared global temporal architecture present in both conditions, within which NoGo trials express condition-specific bifurcations during the early processing phase. This framework highlights the importance of higher order temporal structure in microstate dynamics, revealing aspects of neural computation that remain invisible to conventional microstate metrics.

### Microstate topographies and functional interpretation

The present analysis identified five microstate classes (A–E), four of which corresponded to canonical maps previously described in the literature,^17^ while microstate C displayed a task-specific topography not typically reported in resting-state studies. This suggests that task-related paradigms may recruit configurations specific to cognitive demands, extending beyond the canonical four-microstate solution commonly observed at rest. EEG microstates display specific topographies across all channel arrays, typically interpreted as momentary global states of the brain reflecting the activity of large-scale functional networks within a millisecond range.^16^ These states can arise from resting conditions but also from stimulus presentation,^16^ and their association with resting-state networks identified using fMRI suggests that large-scale networks constitute the neural generators of specific microstate classes.^19^

The three motifs significantly enriched in the NoGo condition during the early phase all pass through microstate A and terminate in microstate D. Microstate E aligned with canonical microstate B, characterized by higher GFP in left frontal regions associated with visual processing and early perceptual integration.^17^^,^^18^ Microstate A resembled canonical microstate C with inverse polarity, characterized by higher GFP in frontal regions typically associated with the default mode network and self-referential processing.^1^ Microstate D aligned with canonical microstate D, characterized by a fronto-central distribution associated with executive function, attentional engagement, working memory, and cognitive control.^14^ Within the NoGo-specific sequences, E may index early attentional processing locked to stimulus presentation, A may reflect visual stimulus detection and attentional reorienting, and D is consistent with early inhibitory processing in fronto-central regions where N2 potentials are generated—a component previously documented in this same dataset.^20^ This interpretation aligns with prior Go/NoGo studies linking similar topographies to motor inhibition.^21^

### Model comparison and conventional microstate metrics

GEE model comparisons revealed no significant sex effects across any of the three conventional microstate metrics. Coverage and lifespan showed no sex-related modulation by either Wald test or information criteria. For occurrences, the Wald test approached but did not reach significance (*p* = 0.074), and although ΔAIC and ΔQIC favored the full model, the absence of a statistically significant Wald test led to exclusion of sex from all final models. These results indicate that neither the proportion of time dominated by each microstate, its mean duration, nor its occurrence rate differed systematically between men and women under the present conditions.

The borderline result for occurrences (*p* = 0.074), combined with the large magnitude of the information criterion differences, suggests that the present sample may have been underpowered to detect a genuine but modest sex effect in microstate transition frequency. This possibility is consistent with prior event-related potential (ERP) research from this same dataset, in which women showed higher N2 amplitudes than men during both Go and NoGo trials in emotional contexts, particularly in frontocentral regions, despite equivalent behavioral performance.^20^ While direct comparison is therefore limited, the convergence between those findings and the present trend suggests that sex differences in inhibitory control—when present—may manifest in the temporal organization of neural dynamics rather than in static microstate properties—a distinction that warrants investigation in larger samples and factorial designs including emotional valence as a variable.

The absence of significant sex-related bias in any metric is nevertheless methodologically useful, confirming that condition-related analyses are not confounded by demographic variation and justifying the use of sex-independent models in all subsequent comparisons.

### Condition-specific motifs: A sequential cascade of inhibitory control

The conditional successor analysis revealed that condition-specific sequential structure is not detectable at the level of marginal motif frequencies or individual transition rates, but emerges when the analysis is conditioned on specific preceding transitions—isolating the branching point at which NoGo trials selectively channel microstate dynamics toward inhibitory states. Three motifs survived FDR correction in the early phase (100–500 ms), all enriched in NoGo: A-C-D (*p*_FDR_ = 0.013), B-A-D (*p*_FDR_ = 0.029), and E-A-D (*p*_FDR_ = 0.040).

The statistical evidence established earlier—significant enrichment of motifs A-C-D, B-A-D, and E-A-D in the NoGo-early phase—supports a sequential interpretation of inhibitory control dynamics. The functional mapping of each transition onto specific cognitive stages draws on the canonical network associations of each microstate (Table 1) and should be understood as a theoretically grounded inference rather than a direct empirical validation.

These three motifs form a functionally coherent set, united by two structural regularities. First, all three motifs pass through microstate A—associated with default mode and visual networks—suggesting that visual stimulus processing constitutes a common entry point into NoGo-specific sequential dynamics. Second, all three motifs terminate in microstate D—associated with executive function and inhibitory control—suggesting D as the common endpoint of inhibitory engagement. A-C-D may represent a route to inhibition proceeding through salience evaluation (C), while E-A-D is consistent with resolution through attentional re-engagement (A) following executive monitoring (E). Notably, the A → D transition—shared by two of the three motifs (B-A-D and E-A-D)—was one of only two bigrams that differed between conditions at the trial level before FDR correction (*p* = 0.021 and *p*_FDR_ = 0.222), consistent with its role as the common effector step toward inhibitory engagement.

This cascade interpretation is supported by the functional associations of each microstate. Microstate D, topographically dominant in fronto-central regions, may reflect activity in the anterior cingulate cortex and lateral prefrontal cortex, structures fundamental to inhibitory control,^22^ and may correspond to the neural substrate of the N2 potential previously documented in this dataset.^20^^,^^23^ Critically, the E → A transition—the entry point into the E-A-D motif—showed no frequency difference between conditions (early phase: *p* = 0.899), confirming that the divergence between Go and NoGo is not in the initiation of this transition but in its resolution: whether the system routes toward D or toward an alternative successor. This positions the conditional successor analysis as methodologically essential, since marginal transition probabilities alone cannot capture the branching structure that distinguishes Go from NoGo dynamics.

The temporal restriction of the E-A-D effect to the early phase (100–500 ms), with no significant E-A-D motif in the late phase, aligns with a reactive control framework in which neural dynamics are rapidly reconfigured following stimulus onset to support perceptual encoding, cognitive evaluation, and the implementation of either response execution (Go) or inhibition (NoGo). Within the dual mechanisms of control framework,^22^ this pattern is consistent with transient, stimulus-triggered engagement rather than sustained anticipatory pre-activation. The critical divergence between conditions occurs during the preparatory and stimulus-locked phases rather than during post-stimulus response execution, challenging stimulus-centric models of cognitive control and supporting the view that flexible early configurations are a primary mechanism through which the brain implements context-appropriate behavior.

This temporal specificity is also consistent with the latency of the N2/P3 inhibitory complex, and with evidence showing that preparatory control states precede and shape response-phase dynamics.^24^

### Shared temporal architecture with condition-specific local structure

The MSA analysis provided a complementary perspective by quantifying global trajectory similarity across individuals within each condition and temporal window. All four cells—Go-early, Go-late, NoGo-early, NoGo-late—showed observed between-subject similarity significantly exceeding their shuffled null distributions (range: 0.333–0.349; all *z* > 11.5 and all *p* < 0.001; OR = 2.60–3.47), confirming that microstate sequences encode non-random temporal organization that cannot be attributed to state-frequency composition alone.

Similarity values were comparable across all four cells, indicating that both Go and NoGo trials maintain a common global temporal architecture throughout the epoch. This shared structure is a general property of task-related neural dynamics rather than a condition-specific phenomenon, and aligns with recent fMRI evidence showing that inhibitory control involves modulation of connectivity patterns within a shared network architecture rather than recruitment of entirely new brain regions.^25^

Within this shared architecture, the group-level ORs revealed a directionally consistent pattern: NoGo conditions yielded higher ORs than Go in both temporal windows (NoGo-early: 3.47 vs. Go-early: 2.64; NoGo-late: 3.17 vs. Go-late: 2.60), suggesting greater relative sequential constraint in NoGo sequences. Within NoGo, the early window showed a higher OR than the late window (3.47 vs. 3.17), whereas Go ORs were virtually identical across windows (2.64 vs. 2.60). Although overlapping confidence intervals preclude formal statistical inference on these differences, this pattern is consistent with stronger local sequential constraint during early NoGo processing—the same window in which the three condition-specific motifs were identified—suggesting that inhibitory demands impose additional temporal regularity on microstate dynamics during early processing.

The logo plots provide a descriptive view of how this structure is organized within the shared global architecture. During the early phase (100–500 ms), the consensus sequences of both conditions include the E-A-D sequence, coinciding temporally with the N200 component documented in this dataset. During the late phase (500–900 ms), Go sequences exhibit repetitive B-C patterns while NoGo sequences display greater configurational variability extending into the P3 window. In contrast to resting-state EEG, where microstate sequences tend to be more stereotyped,^5^^,^^26^ this task-related variability is not noise but signal—representing the brain’s adaptive capacity to generate diverse trajectories in response to varying cognitive demands.

### A dual-level model of microstate organization

The convergence of conditional successor analysis and MSA results support a dual-level model of microstate organization during inhibitory control. At the local level, condition-specific motifs (A-C-D, B-A-D, and E-A-D, all NoGo-enriched) reflect bifurcations in transition dynamics during the early processing window, operating on timescales of hundreds of milliseconds to selectively channel neural trajectories toward inhibitory states. These motifs function as structured control points embedded within a broader temporal sequence. At the global level, MSA demonstrates that both conditions share a common temporal architecture throughout the epoch, with condition-specific differentiation expressed through local sequential patterns rather than wholesale reorganization of trajectory structure.

This dual-level framework reconciles findings from classical microstate metrics with the more nuanced dynamics revealed by sequence-based methods. Coverage and lifespan capture the overall presence of states but are insensitive to temporal order. In contrast, conditional successor analysis and MSA preserve sequential structure, enabling detection of bifurcation points and population-level trajectory similarity that carry functionally relevant information invisible to conventional approaches. The model suggests that cognitive functions do not emerge from specific brain states in isolation but from their temporal organization—how states are sequenced, at which points trajectories diverge, and how that divergence is constrained within a shared global architecture. This perspective aligns with dynamical systems approaches to cognition that emphasize trajectories and attractors rather than static representations.^27^

### Methodological contributions

The present findings contribute to a growing body of work arguing that microstate sequences are better understood in terms of syntax, complexity, and higher order temporal structure than as random strings of discrete states.^5^^,^^28^^,^^29^ This view has been systematically articulated in recent methodological reviews that identify conditional and context-dependent sequence analysis as a central frontier in microstate research,^7^ noting that tools inspired by linguistics and computational biology offer principled means to capture the temporal regularities that conventional metrics overlook.

The present study advances this agenda by integrating two complementary methods from genomics and computational biology. The conditional successor analysis—which isolates the decisional component of sequential dynamics by conditioning on preceding transitions—extends k-mer approaches beyond marginal frequency comparisons toward the detection of context-dependent branching structure. MSA enables quantification of global trajectory similarity at the population level, providing a metric sensitive to overall temporal organization independent of state-frequency composition. Together, these methods parallel genomic approaches used to detect regulatory elements in DNA^30^^,^^31^ and offer a principled framework for linking millisecond-scale neural dynamics to cognitive function.

Future research should pursue several priorities. First, developing substitution matrices for microstate transitions—derived empirically from large normative datasets or theoretically from network connectivity models—would enhance the biological realism of alignment algorithms. Second, extending conditional successor analysis and MSA to other cognitive paradigms—attention, working memory, decision-making, and language processing—would test whether specific bifurcation patterns reliably index particular cognitive operations across paradigms. Third, applying these methods to clinical populations could reveal disruptions in microstate syntax associated with neurological or psychiatric conditions; schizophrenia has been linked to altered microstate dynamics,^32^^,^^33^ and conditional transition metrics may provide more sensitive markers than traditional measures. Fourth, computational modeling using attractor networks, neural mass models, or whole-brain simulations could test mechanistic hypotheses about how local neural interactions give rise to the bifurcation patterns observed empirically.^3^ Finally, combining EEG microstate sequence analysis with fMRI connectivity data would enable direct mapping between electrical states and hemodynamic networks, validating the functional interpretations proposed here.

The broader implications extend beyond inhibitory control. The identification of condition-specific bifurcation motifs during early processing phases supports the importance of proactive control mechanisms across cognitive domains and raises the possibility of differentiating top-down from bottom-up sequential signatures using similar methods. More broadly, the successful translation of genomic sequence analysis tools to neural time-series data exemplifies the value of cross-disciplinary methodological transfer. As neuroscience increasingly grapples with cognition as a temporal process unfolding across hierarchical timescales, conditional sequence analysis, and trajectory-level alignment offer a principled and scalable framework for that endeavor.

This study demonstrates that the temporal organization of EEG microstate transitions captures condition-specific differences in inhibitory control, manifesting as sequential patterning rather than changes in conventional state descriptors. Conditional successor analysis identified three motifs enriched in NoGo-early processing (A-C-D, B-A-D, and E-A-D; all *p*_FDR_ < 0.05), all passing through microstate A and terminating in microstate D, and converging toward fronto-central engagement. MSA revealed that this condition-specific structure is embedded within a shared global temporal architecture present across all conditions and windows. Together, these findings establish sequence-based analysis as a principled framework for linking millisecond-scale brain dynamics to cognitive function, with direct applicability to other paradigms and clinical populations where disruptions in microstate syntax may serve as diagnostic biomarkers.

### Limitations of the study

Several limitations of the present study must be acknowledged. First, the alignment algorithm used fixed scoring parameters (match = +1, mismatch = 0, and gap = −0.5), which may not optimally reflect the functional distances between microstates. Development of biologically informed substitution matrices—analogous to BLOSUM (BLOcks SUbstitution Matrix) or PAM (Point Accepted Mutation) matrices in protein alignment—would improve interpretability and sensitivity by incorporating empirical transition probabilities or theoretical constraints from network models of brain connectivity.^34^ Second, the relatively low GEV (55.1%) for the five-microstate solution indicates that substantial variance remains unexplained. While this is expected for task-locked epochs compared to resting-state benchmarks, future work could explore hierarchical microstate models or continuous representations that better capture cognitive dynamics. Third, the collapsed sequences used in syntax and MSA analyses discard duration information; hybrid approaches incorporating both transition order and state duration could provide a more complete characterization. Fourth, with 30 participants, the present sample provides limited power to detect modest sex effects in microstate metrics; future studies with larger samples should revisit the borderline occurrences result. The moderate split-half reliability of motifs A-C-D, B-A-D, and E-A-D (Spearman-Brown ρ = 0.58–0.64) indicates that their frequency estimates are empirically stable but sensitive to trial sampling, suggesting that larger trial numbers per condition would strengthen the robustness of the syntactic findings in future replication studies.

Finally, the functional interpretation of microstates relies on spatial correlations with canonical maps and inferred network associations; direct validation through simultaneous EEG-fMRI would strengthen claims about neural substrates and enable more precise mapping between electrical states and hemodynamic networks. Additionally, the use of formal meta-criteria for microstate number selection (e.g., cross-validation-based indices) would provide an independent quantitative validation of the K = 5 solution beyond the convergent criteria applied here, and is recommended for future studies.

## Resource availability

### Lead contact

Requests for further information and resources should be directed to and will be fulfilled by the lead contact, Omar Paredes (omar.paredes@academicos.udg.mx).

### Materials availability

This study did not generate new materials or reagents.

### Data and code availability

• Microstate label sequences, group templates and Tables S1, S2, S3, S4, and S5 have been deposited at Mendeley Data and are publicly available as of the date of publication. Mendeley Data: https://doi.org/10.17632/6jcnrr9v58.2. Raw EEG data cannot be deposited in a public repository because not all participants consented to public data sharing; they will be shared by the lead contact upon request.
• All original code has been deposited at Zenodo and is publicly available as of the date of publication. Zenodo: https://doi.org/10.5281/zenodo.23177217. GitHub: https://github.com/Biodigital-Innovation-Lab/cognitive-microstates. The code is a re-implementation of the original analysis, verified against the reported values.
• Any additional information required to reanalyze the data reported in this paper is available from the lead contact upon request.

## Acknowledgments

This work was supported by Secretaría de Ciencia, Humanidades, Tecnología e Innovación (SECIHTI) through a doctoral fellowship to O.C.-G. (CVU 1241368) and a master fellowship to K.G.-D. (CVU 2054918). O.P. would like to sincerely thank Dr. Tere Romero for her valuable discussions and insightful reflections. Her perspective was particularly important in inspiring the analogy of EEG microstates as nucleotides within a dynamic cognitive sequence, providing a meaningful basis for understanding microstate transitions as a form of neural syntax. The authors also thank the participants for their time and cooperation.

## Author contributions

O.C.-G., writing – review and editing, writing – original draft, visualization, validation, software, methodology, formal analysis, conceptualization; K.G.-D., writing – original draft, visualization, software, formal analysis; S.R.-T., writing – original draft, investigation, data curation; H.V.-P., writing – original draft, validation, investigation; J.A.M., writing – original draft, methodology, conceptualization; J.R.-L., writing – original draft, investigation, data curation, conceptualization; R.R.-V., writing – original draft, supervision, project administration, conceptualization; O.P., writing – review and editing, supervision, project administration, methodology, conceptualization

## Declaration of interests

The authors declare no competing interests.

## Declaration of generative AI and AI-assisted technologies in the manuscript preparation process

During the preparation of this work, the authors used Claude Sonnet 4.6 (Anthropic) to improve text fluency and grammar, standardize formatting throughout the manuscript, reduce word count while preserving meaning, and assist in debugging analysis code. Gemini (Google) was used to generate a preliminary sketch of the methodology figure. After using these tools, the authors reviewed and edited the content as needed and take full responsibility for the content of the publication.

## STAR★Methods

### Key resources table

**Table:** 

REAGENT or RESOURCE	SOURCE	IDENTIFIER
**Deposited data**		

EEG Go/NoGo dataset (30 participants)	Ramos-Loyo et al.^20^; available upon reasonable request	N/A
Microstate label sequences, group templates (K = 4–9) and Tables S1, S2, S3, S4, and S5	Mendeley Data	Mendeley Data: https://doi.org/10.17632/6jcnrr9v58.2

**Software and algorithms**		

Analysis code (re-implementation, v1.0.0)	This paper	Analysis code (re-implementation, v1.0.0) | This paper | GitHub: https://github.com/Biodigital-Innovation-Lab/cognitive-microstates; Zenodo: https://doi.org/10.5281/zenodo.23177217
Python 3.11	Python Software Foundation	https://www.python.org/; RRID: SCR_008394
MNE-Python (v1.3)	Gramfort et al.^35^	https://mne.tools; RRID: SCR_005978
Pycrostates (v0.4)	Férat et al.^36^	https://github.com/vferat/pycrostates
statsmodels (v0.14)	Seabold and Perktold^37^	https://www.statsmodels.org; RRID: SCR_016074
BioPython (v1.81)	Cock et al.,^38^	https://biopython.org; RRID: SCR_007173
SciPy (v1.11)	Virtanen et al.^39^	https://scipy.org; RRID: SCR_008058
NumPy (v1.25)	Harris et al.^40^	https://numpy.org; RRID: SCR_008633
pandas (v2.0)	McKinney^41^	https://pandas.pydata.org; RRID: SCR_018214
matplotlib (v3.11)	Hunter^42^	https://matplotlib.org; RRID: SCR_008624
seaborn (v0.13)	Waskom^43^	https://seaborn.pydata.org; RRID: SCR_018132
scikit-learn (v1.9)	Pedregosa et al.^44^	https://scikit-learn.org; RRID: SCR_002577
logomaker (v0.8)	Tareen and Kinney, 2020^45^	https://logomaker.readthedocs.io

**Other**		

Neuronic Medicid 5 EEG system (19-channel, 10–20 system)	Neuronic S.A.	N/A

### Experimental model and study participant details

#### Human participants

Thirty healthy right-handed adults participated in the study: 15 men (mean age 27 years, SD = 2.1) and 15 women (mean age 28 years, SD = 2.7). All participants had completed graduate-level studies and reported no history of neurological or psychiatric disorders. None were under medical treatment or using substances known to affect central nervous system function at the time of data collection. The study protocol was approved by the Ethics Board of the Institute of Neuroscience, University of Guadalajara (approval number: ET012011-92). All participants provided written informed consent prior to participation, in accordance with the Declaration of Helsinki. Participants received no monetary compensation. The data analyzed here were collected under this protocol and were originally described in^20^; the present work is a secondary analysis of the same Go/NoGo recordings.

### Method details

#### Experimental task

Participants performed a Go/NoGo task designed to assess inhibitory control of a prepotent motor response. In each trial, a central arrow was presented at the center of the screen alongside a lateral bar. Participants were instructed to respond (Go condition) when the central arrow matched the lateral bar in both direction and color, and to withhold their response (NoGo condition) when the two stimuli did not match. Stimuli were presented against neutral emotional background images. Each participant completed 20 Go trials and 20 NoGo trials. A complete description of the task parameters, timing, and stimulus presentation sequence is provided in.^20^

#### EEG data acquisition

EEG signals were recorded continuously using a Neuronic Medicid 5 system with 19 electrodes placed according to the international 10–20 system (Fp1, Fp2, F3, F4, F7, F8, C3, C4, T3, T4, T5, T6, P3, P4, O1, O2, Fz, Cz, Pz). Linked earlobes served as the reference during recording, and electrode impedances were maintained below 5 kΩ. Data were sampled at 500 Hz with online bandpass filtering between 0.5 and 50 Hz.

#### EEG preprocessing

Preprocessing was performed using MNE-Python (v1.3;^35^). The data had been cleaned of artifacts prior to delivery. Additional preprocessing steps applied to each recording were: (1) bandpass filtering between 4 and 30 Hz (MNE-Python default FIR filter) to target the theta (4–7 Hz), alpha (8–13 Hz), and beta (13–30 Hz) frequency ranges associated with inhibitory control processes^23^^,^^46^^,^^47^; and (2) re-referencing to the common average, applied to the concatenated record of all trials, which was then split back into 1,300 ms epochs, one per trial. The filtered data were used for clustering and GEV; microstate labeling used the unfiltered epochs. Two non-overlapping temporal windows of interest were defined post hoc: an early phase (100–500 ms relative to epoch onset), reflecting perceptual encoding and early cognitive processing, and a late phase (500–900 ms), reflecting post-response evaluation. These windows were applied consistently across all subsequent sequence analyses. Each window was cut from the 650-sample label series of each trial (early = samples 50–249, late = samples 250–449; 2 ms per sample) and then collapsed (consecutive repeats removed).

#### Microstate analysis

##### Global field power peak extraction

Microstate analysis was performed using the Pycrostates toolbox (v0.4;^36^). For each preprocessed epoch, Global Field Power (GFP) peaks were extracted with a minimum peak distance of 1 sample (2 ms at 500 Hz sampling rate). GFP peaks represent time points of maximal global scalp field strength, which are optimal for microstate template estimation because they minimize interpolation between states.^48^

GEV was computed using the polarity-invariant formula:

$$GEV=\frac{\sum_{t}\left(GFP{\left(t\right)}^{2}\times r{\left(t\right)}^{2}\right)}{\sum_{t}GFP{\left(t\right)}^{2}}$$

where GFP(t) is the global field power at time t, and r(t) is the spatial correlation between the EEG topography at time t and the assigned microstate template.

##### Microstate clustering and group-level aggregation

Clustering was performed using the modified K-means algorithm (ModKMeans) as implemented in Pycrostates. In the first stage, the GFP peaks of each trial (1,200 trials) were submitted separately to ModKMeans, yielding K trial-level maps per trial (6,000 maps for K = 5). Trial-level clustering was performed with the following parameters: random state = 42 (for reproducibility), number of initializations = 100 (default), and polarity ignored (standard practice in microstate research).^1^ In the second stage, the pooled trial-level maps were submitted to a second ModKMeans (same parameters), yielding the group-level templates. For back-fitting, each of the 650 samples of each trial (unfiltered signal) was spatially mean-centred and assigned to the group-level template with the highest absolute spatial correlation (winner-take-all; polarity ignored).

##### Selection of the optimal number of microstates

Clustering solutions were evaluated for K ranging from 4 to 9. The optimal number of microstates was determined using three convergent criteria applied simultaneously: (1) using the 58% benchmark reported for resting-state data^1^ as a reference; task-locked epochs characteristically yield lower GEV values due to increased topographic variability, and the K = 5 solution (GEV = 55.1%) was therefore considered acceptable; (2) demonstrating low spatial overlap between microstate topographies, assessed via pairwise spatial correlation analysis to ensure topographic independence; and (3) exhibiting clear neurophysiological interpretability based on established associations with large-scale brain networks.

Although GEV continued to increase beyond K = 6 (Figure S3), the six-microstate solution (K = 6; GEV = 58.3%) was the first to approach the resting-state benchmark, inspection of its topographies revealed one map with activity concentrated almost exclusively over the right temporal electrode T4 (electrode value: −0.94 μV), constituting an artifact-like pattern inconsistent with physiologically plausible large-scale network dynamics. Furthermore, the spatial correlation matrix for K = 6 showed strong pairwise correlations among microstates D, E, and F (|*r*| = 0.62–0.68), indicating triadic topographic redundancy rather than a meaningful differentiation of network states. A complete comparison of K = 5 and K = 6 topographies and the T4 electrode value analysis is provided in Figure S2.

The five-microstate solution (K = 5; GEV = 55.1%) satisfied all three criteria and was selected for all subsequent analyses. Group templates for K = 4–9 were obtained in a single run of the clustering procedure described above, and the K = 5 templates were used for all analyses (back-fitting, sequences, metrics and their statistics, and canonical correspondence). The templates are deposited in Mendeley Data; re-running the clustering code yields a nearly identical K = 5 solution (spatial correlation >0.99 for every map). The five resulting topographies were spatially distinct and aligned with canonical large-scale network associations reported in prior microstate literature.^1^^,^^17^^,^^19^ Spatial correlation matrices for all K = 4–9 solutions are provided in Figure S3.

##### Microstate sequence extraction

For each trial, each time point was assigned to the microstate template exhibiting the highest polarity-invariant spatial correlation with the current EEG topography (winner-take-all labeling). This procedure yielded a categorical time series of microstate labels per trial. Two complementary sequence representations were derived from this labeling.

• Full sequence: The complete microstate label time series, preserving both state identity and duration information at each time point.
• Collapsed sequence: A reduced representation in which consecutive repetitions of the same microstate label were removed, preserving only the order of state transitions while discarding explicit duration information. This representation was used for all syntax and alignment analyses because the decisional structure of microstate dynamics — which state follows which transition — is fully captured in the transition order, and duration information is separately characterized by the standard microstate metrics (coverage and lifespan). Removing self-repetitions eliminates redundant consecutive assignments that arise from microstate persistence across GFP troughs and do not contribute additional transitional information.

#### Microstate metrics

For each full 1,300 ms epoch, condition (Go, NoGo), and microstate class, three standard parameters were computed from the full (non-collapsed) sequence.

• Coverage: the proportion of the total analysis window during which each microstate was dominant.
• Lifespan (mean duration): the mean duration in milliseconds of each consecutive microstate occurrence.
• Occurrences: the number of discrete appearances of each microstate per epoch.

These metrics were computed individually for each epoch, yielding 6,000 observations per metric (30 participants × 2 conditions × 20 trials × 5 microstates; 5,957 for lifespan, which excludes trials in which a microstate did not occur).

#### Sequence analysis

##### Exhaustive k-mer screen

For each trial, all contiguous subsequences of lengths k = 3, 4, and 5 were extracted from the collapsed sequence. Motif frequencies were compared between Go and NoGo conditions using one-sided Fisher’s exact tests (testing NoGo enrichment) applied to 2 × 2 contingency tables. The Benjamini–Hochberg FDR procedure was applied independently per temporal window and per k-mer length (*α* = 0.05). Only motifs occurring at least five times across both conditions combined were included in the analysis. Effect sizes were quantified using odds ratios (OR > 1 indicating NoGo enrichment) and Cohen’s d computed from trial-level normalized rates.

##### Transition-level analysis

The rate of each of the 20 possible directed bigrams (M_1_→M_2_), excluding self-transitions absent in the collapsed sequences, was computed as the proportion of each transition relative to the collapsed sequence length within each trial. Bigram rates were compared between Go and NoGo conditions using two-sided Mann–Whitney U tests at both the trial level (*n* = 600 per condition; 30 participants × 20 trials) and the subject level (*n* = 30 per condition, averaging each participant’s rates across their 20 trials). Effect sizes were reported as *r* = |*Z*|/√*N*. For the bigram rates (Table S3), *Z* was obtained from the two-sided Mann–Whitney *p* value; for the E→A rate, E-A-D rate and microstate D frequency comparisons, *Z* was computed from U without tie correction, *Z* = (*U* − *n*_1_·*n*_2_/2)/√(*n*_1_·*n*_2_·(*N* + 1)/12).

##### Conditional successor analysis

To isolate the decisional component of microstate transitions—specifically, which state follows a given directed bigram—a conditional successor analysis was performed for each of the 20 directed bigrams. For a given bigram M_1_→M_2_, 2 × 2 contingency tables were constructed comparing the count of a specific triplet completion (M_1_M_2_X) against the count of all alternative successors of that bigram pooled across trials within each condition (Go vs. NoGo), applying one-sided Fisher’s exact tests (testing NoGo enrichment). FDR correction (Benjamini–Hochberg, *α* = 0.05) was applied independently within each bigram’s successor set, yielding 4 comparisons per bigram (excluding the self-transition M_2_→M_2_). The total correction burden across all 20 bigrams was therefore 20 × 4 = 80 comparisons, partitioned into 20 independent FDR families of 4 tests each. This within-bigram correction strategy was adopted to control the error rate at the level of each decisional branchpoint rather than globally, which would have been overly conservative given the conditional independence of bigram contexts. Analyses were conducted separately for the full epoch, early phase (100–500 ms), and late phase (500–900 ms).

##### Pairwise sequence alignment and consensus construction

Multiple Sequence Alignment (MSA) was implemented using BioPython^38^ with the Needleman–Wunsch algorithm (global alignment mode). Scoring parameters were: match = +1, mismatch = 0, gap penalty = −0.5. Pairwise global alignments were computed between the subject-level consensus sequences of every pair of participants within each condition and window (435 pairs per cell). Raw alignment scores were normalized to a [0, 1] similarity index by dividing by the maximum achievable score (length of the shorter sequence × match score). Formula as

$$S=\frac{NW_{score}}{\min\left(\left|s_{1}\right|,\left|s_{2}\right|\right)\cdot m}$$

where *NW*_*score*_ is the alignment score, |*s*_*i*_| denotes sequence length, and *m* corresponds to the match score. Alignment was performed separately for each temporal window (early: 100–500 ms; late: 500–900 ms) and condition (Go, NoGo), yielding four independent alignment sets.

Consensus sequences were derived at three hierarchical levels (Figure S1): (1) a global consensus, defined as the most frequent microstate at each position across all trials within a condition-window combination (*n* = 600 trials per cell); (2) a subject-level consensus, generated from each participant’s 20 trials: the 20 collapsed window sequences were aligned to the median-length sequence by global pairwise alignment (match +1, mismatch −1, gap opening −2, gap extension −0.5), and at each aligned position the most frequent microstate among non-gap characters was retained (support = count of the winning microstate/number of sequences); and (3) a meta-consensus, derived from the 30 subject-level consensus sequences.

For logo plot construction, collapsed sequences from each trial were segmented according to their temporal window boundaries. To enable position-wise comparison across trials and participants, sequences were aligned relative to the onset of the corresponding temporal window (100 ms for early phase, 500 ms for late phase), with each position in the collapsed sequence corresponding to a successive microstate transition from that anchor point. Consensus positions were computed across the 30 subject-level sequences constituting the meta-consensus. Positions with insufficient coverage due to between-subject variability in collapsed sequence length (typically ≤2 subjects absent at that position) were excluded from interpretation and displayed in gray in the logo plots. Logo plots were generated using logomaker,^45^ with letter height proportional to the probability of each microstate at each consensus position. Only microstates reaching a representation threshold of ≥25% at a given position were retained. The plotting code for the logos is not part of the deposited analysis code.

### Quantification and statistical analysis

#### Spatial correspondence with canonical microstate templates

To assess the topographic validity of the empirical five-microstate solution and its relationship to established canonical nomenclature, polarity-invariant spatial similarities (|*r*| × 100) were computed between each empirical microstate map and three external reference sets: Koenig et al. (2002; four maps),^13^ Milz et al. (2016; four maps),^14^ and Artoni et al. (2023; five maps).^15^ Scalp topographies were restricted to the common 19-channel montage, spatially mean-centered, and compared using Pearson correlation across electrodes, with polarity ignored per standard microstate methodology.^1^ Best-match assignments were determined both by maximum |*r*| per empirical map and by globally optimal one-to-one pairing via the Hungarian algorithm, which prevents duplicate canonical assignments. Complete similarity matrices, best-match tables, and one-to-one assignment results for all three reference sets are reported in Data S1 and summarized in Figure S6.

#### Generalized estimating equations

To assess sex effects and condition-level differences in microstate metrics while accounting for within-subject dependencies across repeated trials, Generalized Estimating Equations (GEE) were implemented using statsmodels (v0.14) in Python. An exchangeable working correlation structure was adopted to capture within-subject correlations across trials. For each metric, a full model (metric ∼ condition + microstate + sex + condition:sex) was compared against a reduced model (metric ∼ condition + microstate) using Wald joint tests for sex-related terms and differences in Akaike Information Criterion (ΔAIC; threshold |ΔAIC| > 2) and Quasi-likelihood under the Independence Model Criterion (ΔQIC;^49^). Distribution families and link functions were selected according to the statistical properties of each metric: Coverage (binomial family, logit link), Lifespan (gamma family, log link), and Occurrences (negative binomial family, log link, with overdispersion parameter α estimated via the method of moments; α = 0.125). Trials in which a microstate did not occur (lifespan = 0; 43 of 6,000 observations) were excluded from the lifespan model. ΔAIC was computed from GLM fits with the same model formulas and ΔQIC from the GEE fits. Because coverage sums to 1 across the five microstates in each trial, the condition and sex terms of the coverage model are null by construction.

#### Permutation testing

To determine whether MSA alignment scores reflected genuine temporal structure rather than microstate frequency composition, a permutation-based null model was implemented. For each condition-window combination, sequences were character-shuffled (independently for each sequence, preserving microstate label frequencies while destroying temporal order) and re-aligned over 1,000 iterations (random seed 42), generating a null distribution of mean similarity scores. One-tailed permutation *p*-values were computed as the proportion of null means equal to or exceeding the observed mean. Effect sizes were expressed as z-scores relative to the null distribution. At the group level, observed scores were classified relative to the median of 20 shuffled replicates and compared using Fisher’s exact test (group-level OR, with 0.5 added to each cell and the 95% confidence interval computed on the log scale). At the subject level, each participant’s score was tested against 500 subject-specific shuffles (empirical *p* < 0.05), and the proportion of significant subjects was compared against the 5% expected by chance via a one-tailed binomial test.

#### Multiple comparison correction

Benjamini–Hochberg FDR correction (*α* = 0.05) was applied to all multiple comparison procedures. For the exhaustive k-mer screen, FDR correction was applied independently per temporal window and k-mer length. For the conditional successor analysis, FDR correction was applied within each bigram’s successor set (4 comparisons per bigram; 20 independent families). For transition-level analyses, FDR correction was applied across all 20 directed bigrams within each temporal window and analysis level (trial-level, subject-level). FDR-corrected values are reported as *p*_FDR_; uncorrected values (bigram rates, microstate D frequency, split-half reliability and GEE Wald tests) are reported as *p*.

#### Split-half reliability

To assess the empirical stability of the three FDR-significant motif frequencies (A-C-D, B-A-D, E-A-D), a split-half reliability analysis was conducted by dividing each participant’s 20 NoGo trials into two non-overlapping halves (trials 1–10 vs. 11–20). The rate of a motif in a trial was its number of occurrences divided by the number of possible start positions in the collapsed early-window sequence; mean motif rates were computed per half per participant and correlated across participants using Pearson r, with full-test reliability estimated via the Spearman-Brown correction formula.

## Supplemental information

Document S1. Figures S1–S6

Table S1. Coverage, lifespan, and occurrences of each microstate (A–E) in the K = 5 solution per participant, condition, and trial

Table S2. Counts, percentages, odds ratios, and uncorrected and FDR-corrected *p* values for all 3-, 4-, and 5-mer motifs occurring at least five times in the full epoch, early window, and late window A.) Summary of motifs tested. B.) 3-mers. C.) 4-mers. D.) 5-mers.

Table S3. Mean and standard deviation of the rates of all 20 directed microstate transitions per condition, with Mann-Whitney U statistics, uncorrected and FDR-corrected *p* values (asterisks mark uncorrected *p* < 0.05 only), and effect sizes A.) Full epoch, subject level. B.) Full epoch, trial level. C.) Early window, subject level. D.) Early window, trial level. E.) Late window, subject level. F.) Late window, trial level.

Table S4. One-sided Fisher’s exact tests of NoGo enrichment with FDR correction for the successor of each directed bigram A.) Full epoch. B.) Early window. C.) Late window.

Table S5. Per-trial microstate sequences (full and collapsed; full epoch, early window, and late window) and per-participant consensus sequences with the number of trials, length, and mean support A.) Sequences by trial. B.) Consensus by participant, full epoch. C.) Consensus by participant, early window. D.) Consensus by participant, late window.

Data S1. Complete spatial similarity matrices, best-match assignments, and one-to-one Hungarian assignments between the empirical microstates and three canonical reference sets
