# Supplementary material for: Sequential syntax of EEG microstates captures the temporal dynamics of inhibitory control
Source: iScience. 2026 Nov 3;29(11):118097. doi: 10.1016/j.isci.2026.118097 (PMC13756343; doi:10.1016/j.isci.2026.118097)
Supplement: Supplementary file 1 [file mmc1.pdf]

iScience, Volume *29*

Supplemental information

Sequential syntax of EEG microstates captures the temporal dynamics of inhibitory control

Omar Cano-Garcia, Keith Guzmán-Díaz, Sergio Rivera-Tello, Hugo Vélez-Pérez, J. Alejandro Morales, Julieta Ramos-Loyo, Rebeca Romo-Vázquez, and Omar Paredes

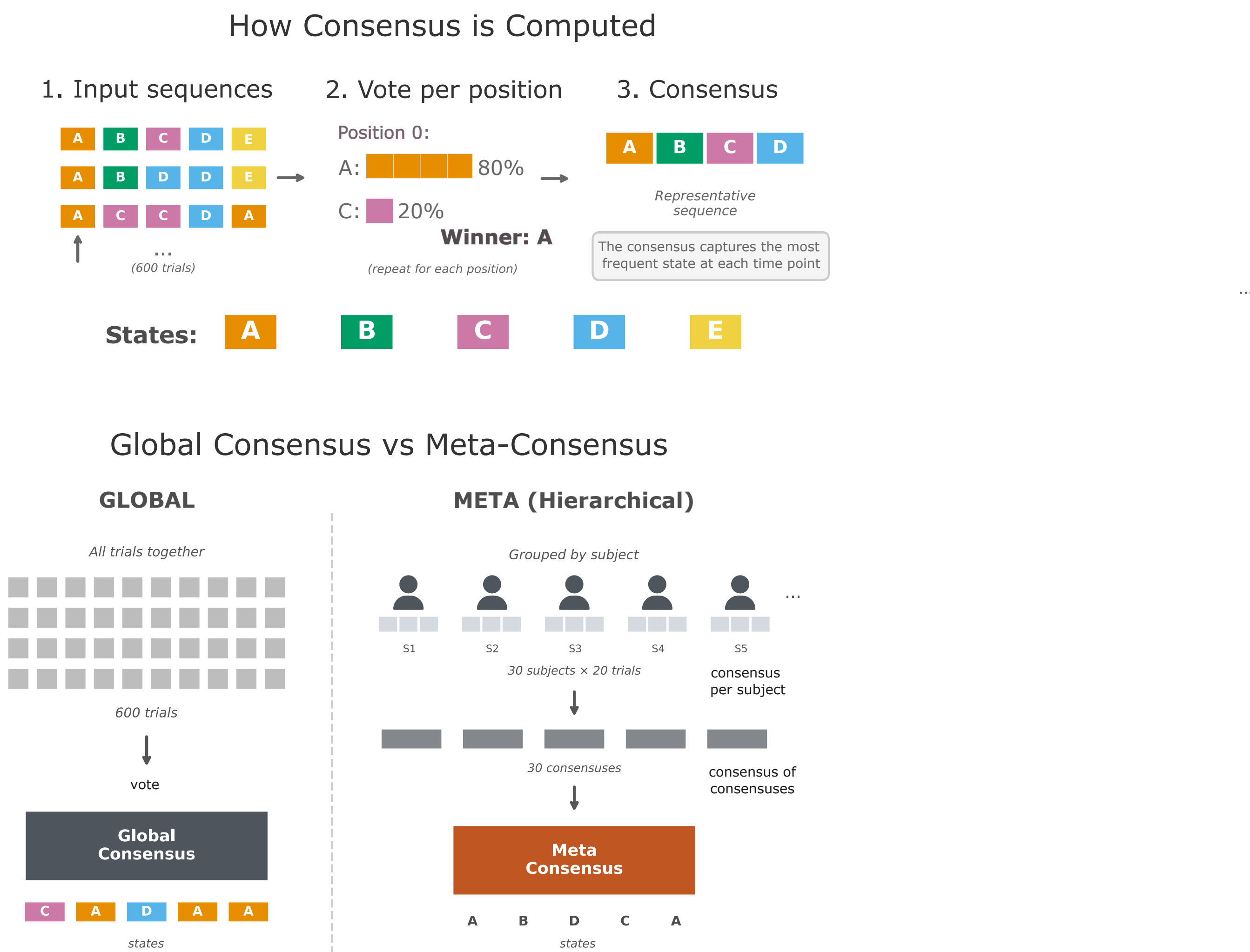

Figure S1: Voting procedure used to derive global and meta-consensus sequences. Schematic of consensus computation. Top: input trial sequences (600 trials per condition and window) are compared position by position, the most frequent microstate wins the vote at each position, and the winners form the representative (consensus) sequence. Bottom: the global consensus is obtained by a single vote across all trials, whereas the meta-consensus is hierarchical, a consensus of the 30 subject-level consensus sequences, each derived from a participant's 20 trials.

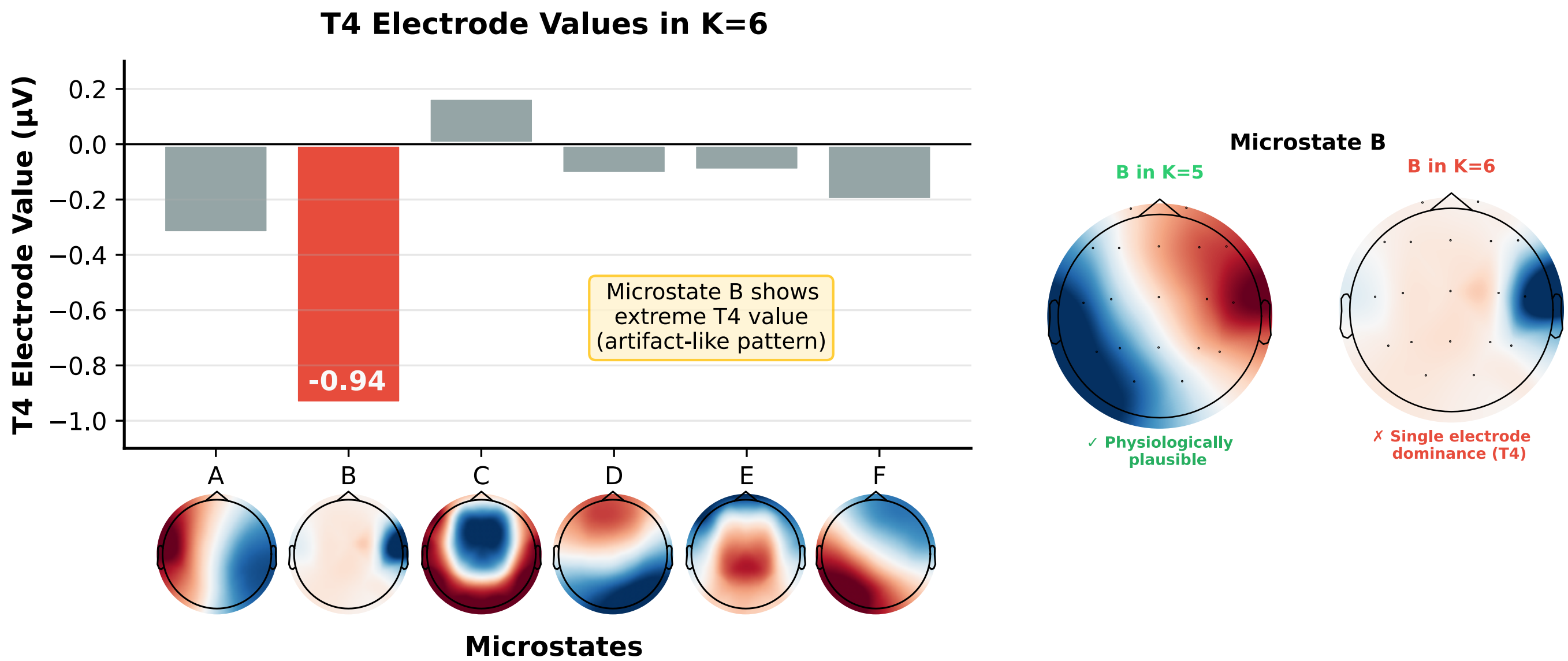

Figure S2: The K = 6 solution contains an artifact-like microstate dominated by electrode T4. Left: T4 electrode value for each of the six microstates (A–F) in the K = 6 solution, with the corresponding scalp topographies shown below the bars. Microstate B shows an extreme T4 value (−0.94 μV, highlighted in red), an artifact-like pattern. Right: topographic comparison of microstate B in the K = 5 solution (physiologically plausible) and in the K = 6 solution (single-electrode dominance at T4).

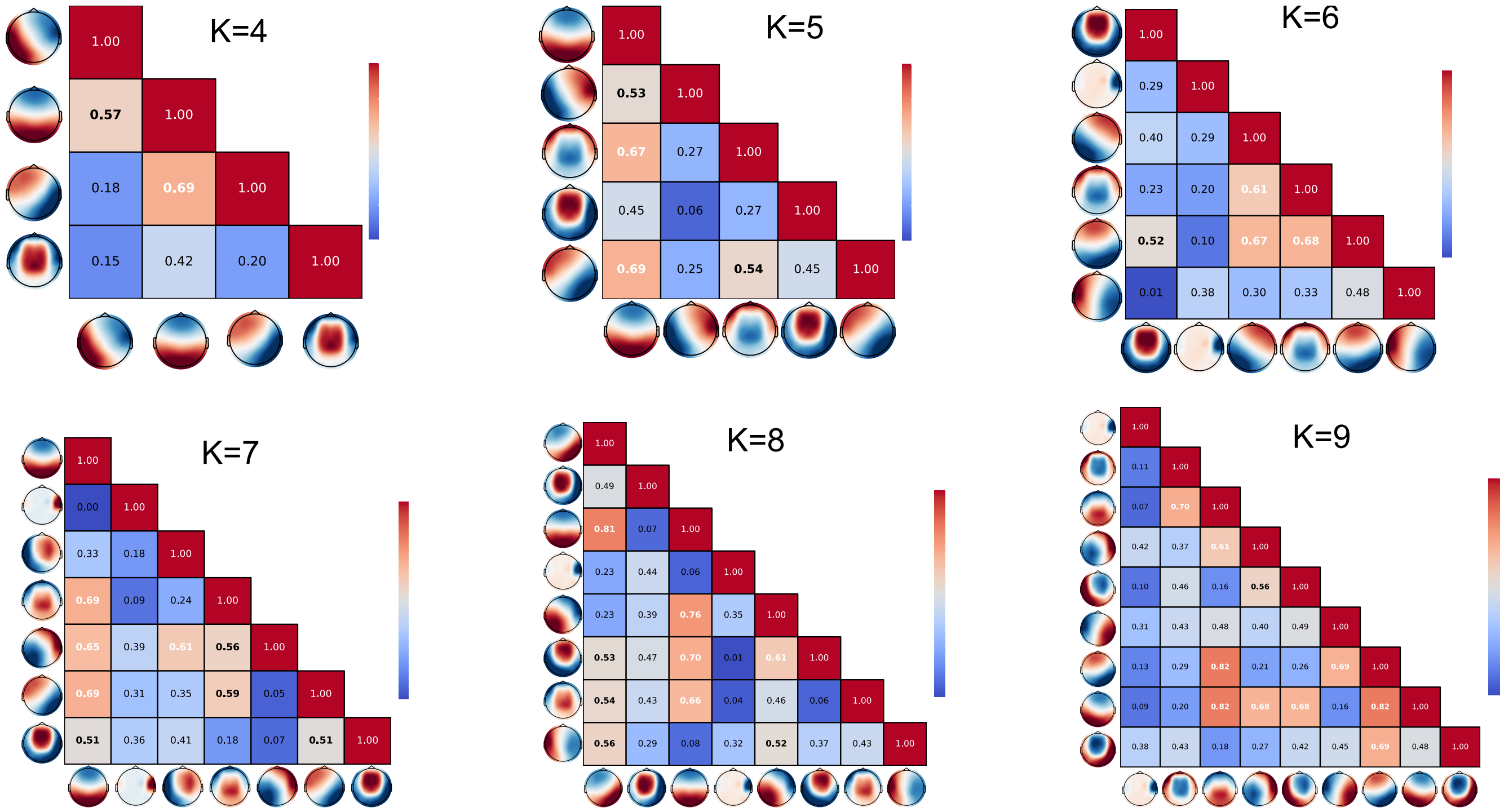

Figure S3: Spatial correlation matrices and topographies for all clustering solutions from K = 4 to K = 9. Each matrix shows the pairwise polarity-invariant spatial correlations among the microstate topographies of one clustering solution (K = 4 to K = 9), with the corresponding scalp maps along the axes. Cell values are spatial correlation coefficients.

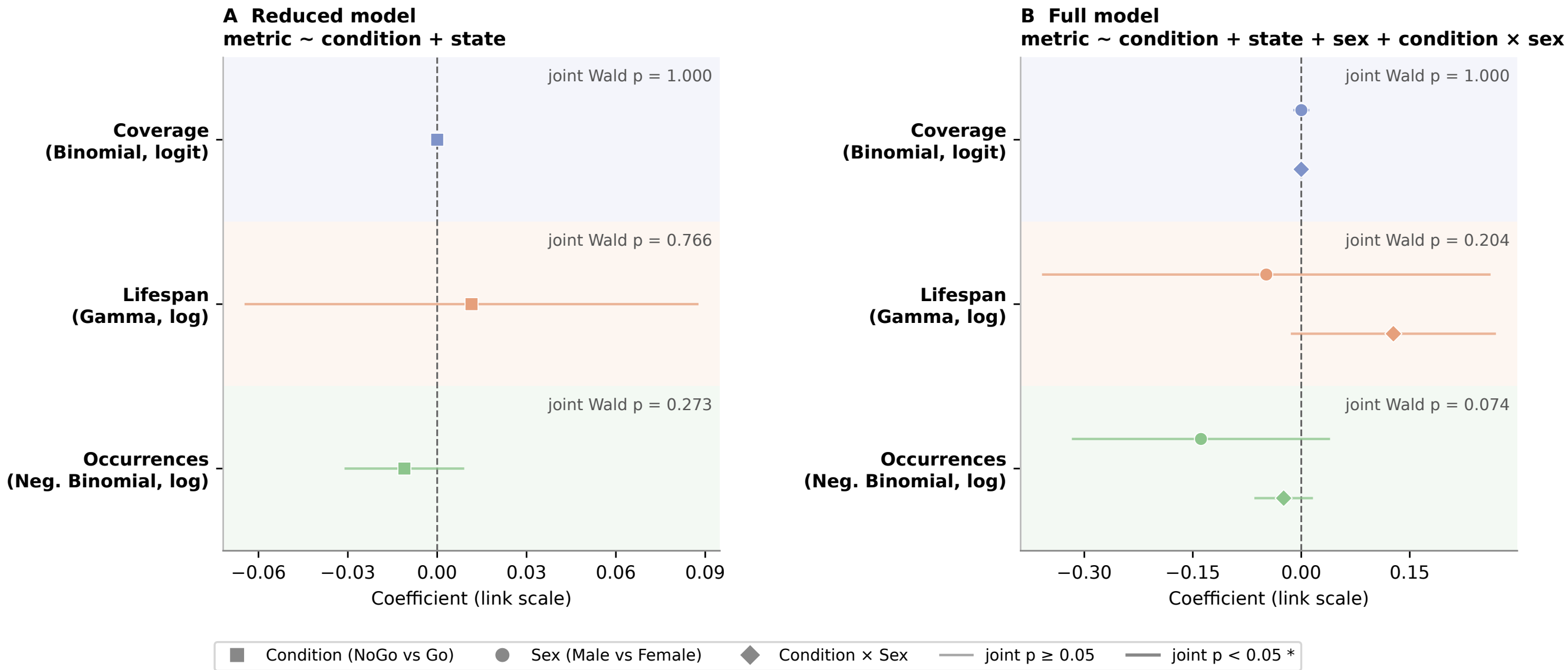

Figure S4: Effects of sex and condition on global microstate metrics estimated with generalized estimating equations (K = 5). A.) Reduced model (metric ~ condition + state). B.) Full model (metric ~ condition + state + sex + condition × sex). Points show coefficients on the link scale with 95% confidence intervals for coverage (binomial, logit), lifespan (gamma, log) and occurrences (negative binomial, log). Squares: condition (NoGo vs. Go); circles: sex (male vs. female); diamonds: condition × sex. Joint Wald *p*-values are shown for each metric; thick lines would indicate joint $p < 0.05$.

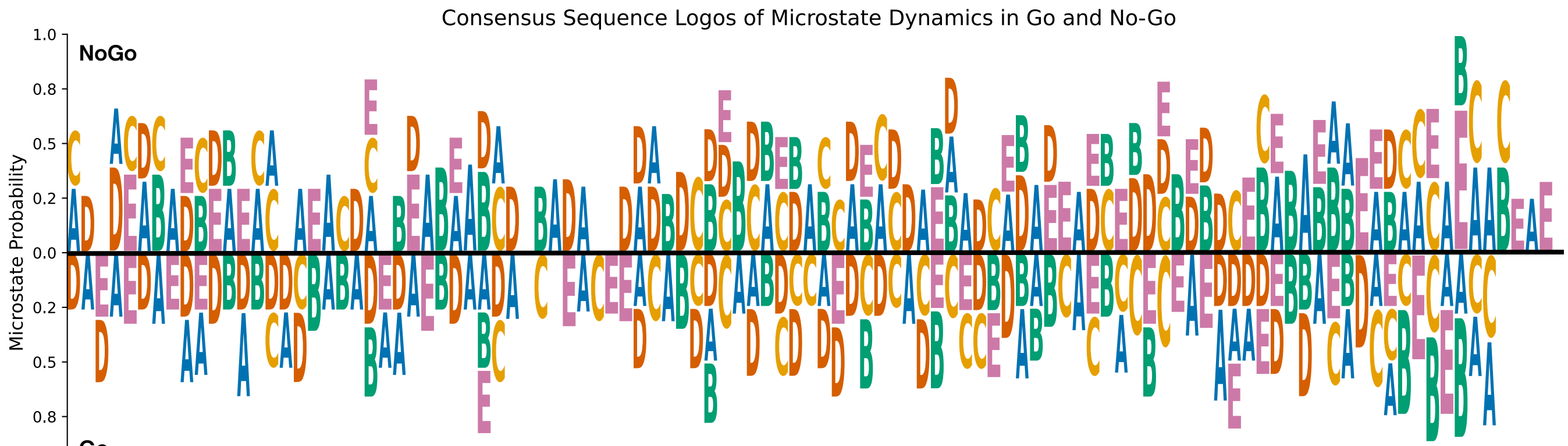

Figure S5: Consensus sequence logos of microstate dynamics in the Go and NoGo conditions across the full epoch. Logo plot of the consensus microstate sequences over the 1,300 ms epoch. The upper half shows the NoGo condition and the lower half the Go condition; letter height indicates the probability of each microstate (A–E) at each consensus position.

A Empirical microstate topographies (K=5, this study)

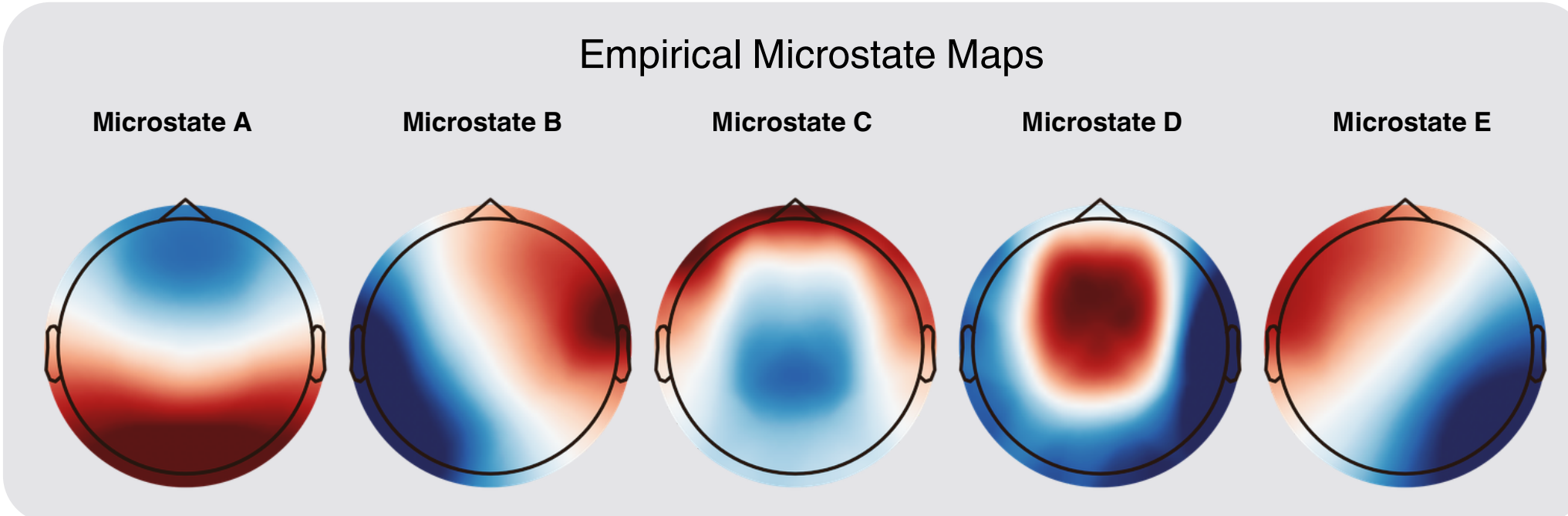

B-D Polarity-invariant spatial similarity matrices (|r| × 100)

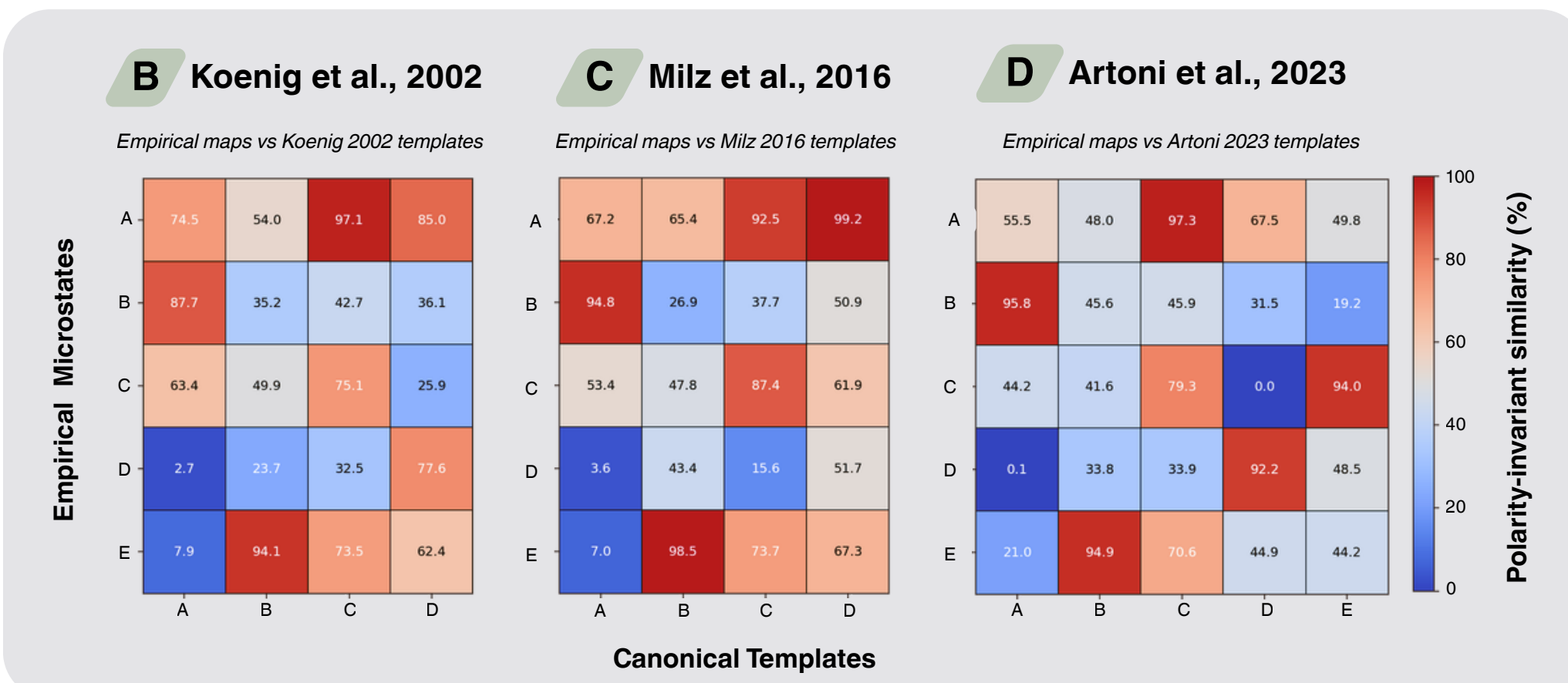

E One-to-one Hungarian assignment — consolidated across reference sets

Empirical Map	Koenig et al. 2002 (4-map)	Milz et al. 2016 (4-map)	Artoni et al. 2023 (5-map)	Functional Association
**A**	**C** |r|=97.1% (inv.) ✓	**D** |r|=99.2% (inv.) ✓	**C** |r|=97.3% (inv.) ✓	Default Mode / Visual networks
**B**	**A** |r|=87.7% ✓	**A** |r|=94.8% ✓	**A** |r|=95.8% ✓	Auditory processing
**C**	unassigned †	**C** |r|=87.4% ✓	**E** |r|=94.0% (inv.) ✓	Task-specific processing
**D**	**D** |r|=77.6% ✓	unassigned †	**D** |r|=92.2% ✓	Executive function
**E**	**B** |r|=94.1% ✓	**B** |r|=98.5% ✓	**B** |r|=94.9% ✓	Visual processing / Attention

✓ one-to-one Hungarian assignment (globally optimal). † Unassigned: K=5 > K=4 canonical maps; mathematically expected. (inv.) inverse polarity; no interpretive consequence (Michel & Koenig, 2018). Teal column (Artoni et al., 2023): complete 5-map assignment, |r| = 92–97%, all 'high'. Yellow: unassigned cells. Similarity thresholds: high ≥80%; moderate 60–79%; low <60%.

Figure S6: Spatial correspondence of the empirical microstates with canonical templates. A.) Scalp topographies of the five empirical microstates (K = 5). B–D.) Polarity-invariant spatial similarity matrices (|*r*| × 100) between the empirical microstates (rows) and the canonical templates (columns) of Koenig et al. (2002) (B), Milz et al. (2016) (C) and Artoni et al. (2023) (D). E.) One-to-one Hungarian assignment consolidated across the three reference sets; check marks indicate the globally optimal assignment, † indicates unassigned maps (K = 5 > K = 4 canonical maps; mathematically expected), and (inv.) indicates inverse polarity.
