# Supplementary material for: Sequential syntax of EEG microstates captures the temporal dynamics of inhibitory control
Source: iScience. 2026 Nov 3;29(11):118097. doi: 10.1016/j.isci.2026.118097 (PMC13756343; doi:10.1016/j.isci.2026.118097)
Supplement: Supplementary file 2 [file mmc2.pdf]

Data S1 — Canonical Microstate Template Matching

Overview

This supplementary document reports the polarity-invariant spatial correlations between the five empirical EEG microstate topographies and three external canonical reference sets, providing a cross-study validation of the K=5 solution. All results reported here are also summarized visually in **Figure S6**.

Methods

Reference template sets

Three canonical reference sets were used:

1. **Koenig et al. (2002):** Four canonical microstate maps (A–D) derived from resting-state EEG in healthy adults (N = 496). Reference: Koenig, T., et al. (2002). NeuroImage, 16(1), 81–88. https://doi.org/10.1006/nimg.2002.1070

2. **Milz et al. (2016):** Four canonical microstate maps (A–D) from resting-state EEG with standardized preprocessing (N = 164). Reference: Milz, P., et al. (2016). NeuroImage, 128, 23–33. https://doi.org/10.1016/j.neuroimage.2015.08.023

3. **Artoni et al. (2023):** Five canonical microstate maps (A–E) from resting-state EEG providing the first large-sample five-class solution (N = 205). Reference: Artoni, F., et al. (2023). NeuroImage, 277, 120196. https://doi.org/10.1016/j.neuroimage.2023.120196

Spatial similarity computation

For each pair of empirical and canonical microstate maps, scalp topographies were restricted to the common 19-channel montage (Fp1, Fp2, F3, F4, F7, F8, C3, C4, T3, T4, T5, T6, P3, P4, O1, O2, Fz, Cz, Pz; 10–20 system), spatially mean-centered, and compared using Pearson correlation across electrodes. Similarity is reported as $|r| \times 100$ (polarity-invariant), with signed r retained to document polarity orientation. This approach follows Michel & Koenig (2018) in which microstate polarity is considered an arbitrary artifact of the clustering algorithm and not a meaningful difference between maps.

Best-match and one-to-one (Hungarian) assignment

Two assignment strategies were applied:

Best-match assignment: Each empirical map is matched to the canonical map with the highest $|r|$, independently of other assignments. Multiple empirical maps may match the same canonical map.

One-to-one (Hungarian) assignment: The Hungarian algorithm (Kuhn, 1955) identifies the globally optimal one-to-one pairing that maximizes total similarity while ensuring no canonical map is assigned to more than one empirical map. When the number of empirical maps (K=5) exceeds the number of canonical maps (K=4 in Koenig and Milz sets), one empirical map necessarily remains unassigned. This unassignment is mathematically expected and informationally meaningful: it identifies the empirical map that best functions as the "fifth class" not represented in four-map solutions. Reference: Kuhn, H.W. (1955). Naval Research Logistics Quarterly, 2(1–2), 83–97.

Similarity thresholds

Following Artoni et al. (2023): high ≥ 80%; moderate 60–79%; low/ambiguous < 60%.

Results

Koenig et al. (2002) — Full spatial similarity matrix

Values represent polarity-invariant spatial similarity (|r| × 100). Green shading: ≥80% (high). Blue shading: <60% (low). Rows: empirical maps (A–E = MS1–MS5). Columns: canonical maps.

Empirical map	Koenig 2002 A	Koenig 2002 B	Koenig 2002 C	Koenig 2002 D
**A (MS1)**	74.54	54.00	**97.15**	**85.05**
**B (MS2)**	**87.73**	35.23	42.69	36.05
**C (MS3)**	63.38	49.87	75.09	25.89
**D (MS4)**	2.68	23.73	32.52	77.59
**E (MS5)**	7.85	**94.15**	73.53	62.37

Best-match assignment — Koenig et al. 2002

Empirical map	Best canonical match	Similarity (%)	Signed r	Interpretation	Second best (%)
**A (MS1)**	Koenig 2002 C	97.15	−0.97	high	Koenig 2002 D 85.05%
**B (MS2)**	Koenig 2002 A	87.73	+0.88	high	Koenig 2002 C 42.69%
**C (MS3)**	Koenig 2002 C	75.09	+0.75	moderate	Koenig 2002 A 63.38%
**D (MS4)**	Koenig 2002 D	77.59	+0.78	moderate	Koenig 2002 C 32.52%
**E (MS5)**	Koenig 2002 B	94.15	+0.94	high	Koenig 2002 C 73.53%

† Under one-to-one Hungarian assignment: MS1 (A) → Koenig C; MS2 (B) → Koenig A; MS4 (D) → Koenig D; MS5 (E) → Koenig B. MS3 (C) remains unassigned (K=5 > K=4).

Milz et al. (2016) — Full spatial similarity matrix

Empirical map	Milz 2016 A	Milz 2016 B	Milz 2016 C	Milz 2016 D
**A (MS1)**	67.19	65.45	**92.49**	**99.24**
**B (MS2)**	**94.78**	26.90	37.68	50.86
**C (MS3)**	53.38	47.77	**87.36**	61.92
**D (MS4)**	3.58	43.35	15.55	51.72
**E (MS5)**	7.02	**98.52**	73.66	67.29

† Under one-to-one Hungarian assignment: MS1 (A) → Milz D; MS2 (B) → Milz A; MS3 (C) → Milz C; MS5 (E) → Milz B. MS4 (D) remains unassigned (low/ambiguous best-match: 51.72%).

Artoni et al. (2023) — Full spatial similarity matrix

Empirical map	Artoni 2023 A	Artoni 2023 B	Artoni 2023 C	Artoni 2023 D	Artoni 2023 E
**A (MS1)**	55.50	47.96	**97.28**	67.51	49.79
**B (MS2)**	**95.77**	45.59	45.90	31.46	19.17
**C (MS3)**	44.20	41.63	79.30	0.03	**94.04**
**D (MS4)**	0.08	33.84	33.89	**92.23**	48.48
**E (MS5)**	20.97	**94.94**	70.57	44.92	44.23

Under one-to-one Hungarian assignment: MS1 (A) → Artoni C; MS2 (B) → Artoni A; MS3 (C) → Artoni E; MS4 (D) → Artoni D; MS5 (E) → Artoni B. All five empirical maps are assigned (complete solution; K=5 = K=5).

Consolidated one-to-one Hungarian assignment (see Figure S6, Panel E)

Empirical Map	Koenig et al., 2002 (4-map)	Milz et al., 2016 (4-map)	Artoni et al., 2023 (5-map)	Functional association
**A (MS1)**	C |r|=97.1% (inv.) ✓	D |r|=99.2% (inv.) ✓	C |r|=97.3% (inv.) ✓	Default Mode / Visual networks
**B (MS2)**	A |r|=87.7% ✓	A |r|=94.8% ✓	A |r|=95.8% ✓	Auditory processing
**C (MS3)**	***unassigned †***	C |r|=87.4% ✓	E |r|=94.0% (inv.) ✓	Task-specific processing
**D (MS4)**	D |r|=77.6% ✓	***unassigned †***	D |r|=92.2% ✓	Executive function
**E (MS5)**	B |r|=94.1% ✓	B |r|=98.5% ✓	B |r|=94.9% ✓	Visual processing / Attention

✓ one-to-one Hungarian assignment (globally optimal). † Unassigned: K=5 > K=4 canonical; expected by design. (inv.) = inverse polarity (signed r < 0). Teal = Artoni et al. 2023 column (complete 5-map assignment). Yellow = unassigned cells. Similarity thresholds: high ≥80%; moderate 60–79%; low <60%.

Interpretation

Microstates A, B, and E: high-confidence canonical correspondence

Microstate A corresponds to the symmetric anterior–posterior class, associated with Default Mode / Visual networks (Koenig C: 97.1%; Milz D: 99.2%; Artoni C: 97.3%) consistently across all three reference sets, including a clear one-to-one Hungarian assignment in all three cases. Microstate B corresponds to the right-frontal-to-left-posterior diagonal class, associated with auditory processing (Koenig A: 87.7%; Milz A: 94.8%; Artoni A: 95.8%), and Microstate E to the left-frontal-to-right-posterior diagonal class, associated with visual processing and attention (Koenig B: 94.1%; Milz B: 98.5%; Artoni B: 94.9%). All three constitute high-confidence canonical correspondences.

Microstate D: canonical assignment confirmed by Artoni et al. (2023)

Microstate D corresponds to the fronto-central class, associated with executive function (Artoni D: 92.2%, high). Its correspondence with four-map solutions is less definitive (Koenig D: 77.6%, moderate; Milz D: 51.7%, low/ambiguous; additionally, the Milz assignment leaves D unassigned under one-to-one constraint). The divergence between reference sets reflects known differences in the "D" map across canonical studies, not a weakness of the empirical map, and is resolved by the five-map Artoni solution.

Microstate C: identification as the task-specific fifth class

Microstate C shows its strongest correspondence with Artoni 2023 E (94.0%, high) — the fifth canonical class that has no equivalent in four-map solutions. Under Koenig (four maps), C remains unassigned in the Hungarian algorithm; under Milz, it is assigned to Milz C (87.4%) but leaves MS4 (D) unassigned. The pattern of unassignment in four-map references combined with high-confidence assignment to Artoni's fifth class (Artoni E) provides convergent evidence that Microstate C represents a task-specific processing state not captured by canonical four-class resting-state solutions, and provides empirical justification for the K=5 solution over K=4.

Significance of the complete Artoni et al. (2023) assignment

The Artoni et al. (2023) five-map solution is the only reference that yields a complete, unambiguous one-to-one assignment for all five empirical maps, with all similarities ≥ 92% and all rated "high." This constitutes the strongest available cross-study validation of the K=5 solution and confirms that the five empirical microstate topographies are not arbitrary clustering outcomes but correspond to established, reproducible scalp field patterns identified independently in large resting-state samples.

References

Artoni, F., Merk, T., Schneider, J., Tiber, V., Sheller, J., Blankertz, B., & König, P. (2023). Microstate analysis of resting-state EEG: A standardized template for large-scale data. NeuroImage, 277, 120196. https://doi.org/10.1016/j.neuroimage.2023.120196

Koenig, T., Prichep, L., Lehmann, D., Sosa, P. V., Braeker, E., Kleinlogel, H., Isenhart, R., & John, E. R. (2002). Millisecond by millisecond, year by year: Normative EEG microstates and developmental stages. NeuroImage, 16(1), 81–88. https://doi.org/10.1006/nimg.2002.1070

Kuhn, H. W. (1955). The Hungarian method for the assignment problem. Naval Research Logistics Quarterly, 2(1–2), 83–97.

Michel, C. M., & Koenig, T. (2018). EEG microstates as a tool for studying the temporal dynamics of whole-brain neuronal networks: A review. NeuroImage, 180, 577–593. https://doi.org/10.1016/j.neuroimage.2017.11.062

Milz, P., Faber, P., Lehmann, D., Koenig, T., Kochi, K., & Pascual-Marqui, R. D. (2016). The functional significance of EEG microstates — Associations with modalities of thinking. NeuroImage, 128, 23–33. https://doi.org/10.1016/j.neuroimage.2015.08.023
